# Parental drinking and adverse outcomes in children: A scoping review of cohort studies

**DOI:** 10.1111/dar.12319

**Published:** 2015-08-31

**Authors:** Ingeborg Rossow, Lambert Felix, Patrick Keating, Jim McCambridge

**Affiliations:** ^1^ Norwegian Institute for Alcohol and Drug ResearchOsloNorway; ^2^ Faculty of Public Health and PolicySchool of Hygiene and Tropical MedicineLondonUnited Kingdom; ^3^ Department of Health SciencesUniversity of YorkYorkUnited Kingdom

**Keywords:** alcohol, harm to others, parental drinking, children, scoping review

## Abstract

**Introduction and Aims:**

There is a growing interest in measuring alcohol's harms to people other than the drinker themselves. ‘Children of alcoholics’ and foetal alcohol spectrum disorder have received widespread attention. Less is known about how children are affected by post‐natal exposure to parental drinking other than alcohol abuse/dependence. In this scoping review, we aim to assemble and map existing evidence from cohort studies on the consequences of parental alcohol use for children, and to identify limitations and gaps in this literature.

**Design and Methods:**

Systematic review methods were used. Electronic databases were searched (1980 to October 2013) and a total of 3215 abstracts were screened, 326 full text papers examined and 99 eligible for inclusion according to selection criteria including separation of exposure and outcome measurement in time and report of a quantitative effect size.

**Results:**

The main finding is the large literature available. Adolescent drinking behaviour was the most common outcome measure and outcomes other than substance use were rarely analysed. In almost two of every three published associations, parental drinking was found to be statistically significantly associated with a child harm outcome measure. Several limitations in the literature are noted regarding its potential to address a possible causal role of parental drinking in children's adverse outcomes.

**Discussion and Conclusions:**

This study identifies targets for further study and provides a platform for more targeted analytic investigations which ascertain risk of bias, and which are capable of considering the appropriateness of causal inferences for the observed associations. [Rossow I, Felix L, Keating P, McCambridge J. Parental drinking and adverse outcomes in children: A scoping review of cohort studies. *Drug Alcohol Rev* 2016;35:397–405]

## Introduction

Alcohol use may harm not only the individual drinker but also the lives of their partners, families, friends, work colleagues and their communities. While the global burden of disease estimates all deaths and disability adjusted life years lost, it may underestimate the aggregate harms caused by alcohol use, as it does not comprehensively measure all of the harms to others [1]. Moreover, most of the literature on the negative effects of alcohol use has focused on the direct harms to drinkers' health and, thus, much less emphasis has been placed on measuring the harms to families and the wider social costs of alcohol use. However, there is a growing interest in measuring these harms to others or ‘externalities’ or ‘collateral damage’, or ‘second‐hand’ effects of alcohol use [2, 3, 4, 5, 6, 7, 8].

In particular, numerous studies have examined the effects of prenatal alcohol exposure [9, 10] and the effects on children living with ‘alcoholics’ or parents with serious and long term alcohol problems. The latter have found evidence that these children are more prone to later adverse outcomes in a broad range of areas, such as substance misuse, behavioural problems and poorer physical and mental health [11, 12, 13]. However, the elevated risk observed in these studies cannot be interpreted as causal effects of parental drinking. Serious and long term alcohol problems are often embedded in a nexus of problems relating to mental health, unemployment, poverty, housing, family and social networks, which are likely to impact on the health and well‐being of children [14], thus hampering valid inferences of causality. Moreover, behavioural resemblance among parents and children may result also from shared genes [15]. Adverse effects of parental heavy drinking *per se* are therefore difficult to disentangle from the adverse effects of other factors.

From the literature on alcohol's direct harms to drinkers, there is evidence that volume of drinking is linked to most disease outcomes through specific dose–response relationships and that harmful effects of drinking are observed also at lower drinking levels [16]. Although the heaviest drinkers are much more likely to experience alcohol related harms, they do not account for all or even most, of the alcohol burden. Particularly, with respect to acute alcohol related harms that are typically seen in relation to heavy episodic drinking, it is often found that most of the harms are attributable to those who are otherwise light and moderate drinkers [16, 17].

When it comes to alcohol's harms to others, much less is known about how children are affected by patterns of alcohol consumption other than clinically diagnosed alcohol problems, including drinking at low risk levels and heavy episodic or binge drinking. In the UK alone, 30% of children are estimated to live with an adult binge drinker, so it is obviously important to better understand how children may be impacted by parental drinking [12]. As well as benefits to scientific understanding, addressing these issues is of likely policy significance. For instance, growing evidence of the effects of passive smoking, a form of harm to others, was a key component in changing policy and practice to denormalise tobacco use [18]. This is because it changes the basis of societal interest in the autonomous behaviour of individuals into harms caused to other people, and protecting children, in particular, from harms is widely accepted as a core concern of social policy.

Systematic reviews are research designs capable of summarising and evaluating existing data. Scoping reviews are systematic reviews that are used to assess the extent of a body of literature available on a particular topic and to summarise and disseminate research findings, usually in order to ensure that further research in that area is a beneficial addition to world knowledge [19, 20, 21]. In addition to exploring the extent of the literature in a particular domain and summarising findings, scoping reviews are used to identify research gaps [22]. Scoping reviews may therefore help to identify a more specific research question, based on what was already known or not known [22].

Although there are systematic reviews in related areas [23, 24], neither has had a primary focus on the breadth of consequences for children, and both have more precise foci and mechanistic orientations. Literature searches in MedLine and Google Scholar (October 2014) identified some scoping reviews related to alcohol use [25, 26], but none were found on parental drinking's possible harmful effects. This scoping review thus offers the first summary of available evidence from cohort studies of parental drinking and child outcome, both in terms of study characteristics and principal findings. It is designed to provide a platform for more in‐depth analytic reviews and aetiological investigations. By design, it is an ‘apples and oranges’ review encompassing heterogeneity in outcomes and other study characteristics.

The overarching aim of this scoping review is thus to assemble and map existing evidence from cohort studies on the consequences of parental alcohol use for children. This includes investigation of whether parental drinking—including hazardous and harmful consumption and excluding clinically diagnosed alcohol problems and prenatal exposure—impacts on children's behaviour and well‐being, including substance use and other health risk behaviours, their mental health, and educational and social outcomes. The specific objectives were (i) to identify the extent and range of studies on this subject and to summarise data on studied outcomes in children as a possible consequence of parental alcohol use; and (ii) to identify limitations and gaps in this literature as a basis for further studies of the possible consequences of parental alcohol use.

## Methods

### Search strategy

We searched five electronic databases: MEDLINE; EMBASE; PsycINFO; Global Health; and Web of knowledge, with the last searches being undertaken on 16 October 2013. One author (P. K.) performed both backward and forward searches to identify any studies that we might have missed [27]. For backward searching, we checked the bibliographies of included studies, while for forward searching, we used Google Scholar and Science Citation Index to identify subsequent citations of the included studies. We contacted 10 experts to identify additional studies and six of these responded. The database search strategy was devised to include terms across parental alcohol use, children and study design domains. Box 1 presents the search strategy that was used for PsycINFO and this was adapted in minor ways for other databases.

Box 1 **Search strategy used for PsycINFO**

**Table:** 

A. Parental drinking	B. Children	C. Study design
1 exp drinking behavior/ (35750) 2 exp alcohol consumption/ (68521) 3 [(heav* drink* or harmful drink* or hazard* drink* or binge drink* or risk* drink* or alcohol drink* or drunk* or alcohol* intoxicat* or drinking rate or alcohol abuse or alcohol misuse or alcohol consum* or alcohol drink*) adj5 (parent* or mother or father or paternal or maternal or guardian* or custodian*)].ti,ab. (1339) 4 [(alcohol* or beer or wine or spirit* or drink) adj3 (unit or consum* or intake or binge or use*)].ti,ab. (85962) 5 exp parent/ (180672) 6 ‘parent*’.ti,ab. (363124) 7 ‘mother*’.ti,ab. (204876) 8 ‘father*’.ti,ab. (40885) 9 paternal.ti,ab. (19220) 10 maternal.ti,ab. (211555) 11 ‘guardian*’.ti,ab. (5480) 12 ‘custodian*’.ti,ab. (312) 13 5 or 6 or 7 or 8 or 9 or 10 or 11 or 12 (733620) 14 [(alcohol* or beer or wine or spirit* or drink) adj3 (unit or consum* or intake or binge or use*) adj5 (parent or ‘parent*’ or ‘mother*’ or ‘father*’ or paternal or maternal or ‘guardian*’ or ‘custodian*’)].ti,ab. (2251) 15 1 or 2 (95088) 16 13 and 15 (6048) 17 3 or 14 or 16 (7196)	18 child/ (1951853) 19 exp adolescent/ (1295524) 20 exp student/ (72698) 21 exp youth/ (24580) 22 early adult.ti,ab. (1287) 23 ‘child*’.ti,ab. (1329505) 24 offspring.ti,ab. (55611) 25 ‘adolescen*’.ti,ab. (216763) 26 ‘famil*’.ti,ab. (913984) 27 ‘juvenil*’.ti,ab. (74354) 28 progeny.ti,ab. (28575) 29 ‘girl*’.ti,ab. (151194) 30 ‘boy*’.ti,ab. (157382) 31 child behavio$r.ti,ab. (4307) 32 ‘teenage*’.ti,ab. (19343) 33 ‘young adult*’.ti,ab. (71575) 34 youth.ti,ab. (40646) 35 ‘child parent relation* ’.ti,ab. (115) 36 ‘pubescen*’.ti,ab. (1855) 37 high school.ti,ab. (21575) 38 ‘teen*’.ti,ab. (26704) 39 young women.ti,ab. (20795) 40 young men.ti,ab. (13344) 41 ‘young male*’.ti,ab. (12682) 42 ‘young female*’.ti,ab. (7858) 43 ‘student*’.ti,ab. (213019) 44 young people.ti,ab. (22012) 45 ‘minor*’.ti,ab. (268062) 46 ‘kid*’.ti,ab. (453316) 47 ‘underage*’.ti,ab. (819) 48 ‘puber*’.ti,ab. (39478) 49 22 or 23 or 24 or 25 or 26 or 27 or 28 or 29 or 30 or 31 or 32 or 33 or 34 or 35 or 36 or 37 or 38 or 39 or 40 or 41 or 42 or 43 or 44 or 45 or 46 or 47 or 48 (3367379) 50 18 or 19 or 20 or 21 or 49 (4645094)	51 exp cohort study/ (157307) 52 exp longitudinal study/ (65016) 53 exp prospective study/ (249464) 54 exp retrospective study/ (341641) 55 51 or 52 or 53 (434085) 56 55 not 54 (399896) 57 ‘cohort stud*’.ti,ab. (102965) 58 ‘prospective stud*’.ti,ab. (150172) 59 follow up.ti,ab. (818842) 60 ‘panel stud*’.ti,ab. (1236) 61 ‘retrospective stud*’.ti,ab. (112662) 62 57 or 58 or 59 or 60 (1015159) 63 [(‘cohort stud*’ or ‘prospective stud*’ or follow up or ‘panel stud*’) not ‘retrospective stud*’).ti,ab. (988089) 64 56 or 63 (1211062)

65 A (17) and B (50) and C (64) = 1111.

### Selection criteria

Prospective cohort studies offer the highest quality observational evidence available. We sought studies that followed families or individuals of interest over a period of time, having at least two data collection points. Exposure data collection was required to precede outcome data collection in time, meaning studies where both the exposure and the outcome data were measured at the same time were excluded. Retrospective cohort studies and other types of observational studies were also excluded. We included studies published in English language peer‐reviewed journals from 1980 onwards.

The exposure measure of parental alcohol use could be obtained from either parent, children or another source such as official records. For this scoping review, we did not apply measurement quality criteria nor set any lower alcohol consumption threshold. Participants included both parents and children from general population samples; those from ‘special populations’ who may have distinct exposure–outcome relationships were excluded. Parental data included that from both or either parent, and for biological or non‐biological parents. We excluded studies where parental drinking was measured with clinical instruments designed for diagnosis (ICD/DSM) or assessment of severity of alcohol problems abuse or dependence, or by brief screening tools designed to identify alcohol dependence or ‘alcoholics’. Studies which assessed only alcohol consumption in parents or consumption plus problems were included, as were problem measures not derived from ICD/DSM criteria as they were judged likely to assess less severe forms and levels of problems. Studies in which the only parental alcohol data were maternal alcohol use measured during pregnancy were excluded.

We imposed no restrictions on outcomes for children, thus including substance use, behavioural and any other health or psychosocial outcomes. These could be assessed at any point in time including in adulthood. We required a quantitative measure of the size of the effect of parental alcohol use on outcomes in children.

We identified 3880 records from searching five electronic databases namely EMBASE, Global Health, Medline, PsycINFO and Web of Science (see Box 1 for the search strategy used in PsycINFO). These records were directly exported into the reference management software EndNote X7 [27]. A total of 264 additional records were identified through other sources such as forward and backward citation searches. We then removed duplicate records of the same report, resulting in 3215 records for the first phase of screening. Two authors (P. K. and L. F.) independently applied the inclusion criteria to the titles and abstracts of each record to examine their inclusion. Both authors agreed to include 326 records as potentially relevant studies and retrieved their full texts. Once again, the same two authors (P. K. and L. F.) independently examined the full texts to assess their eligibility against the screening criteria. The third author (J. M.) assessed any papers where there was any uncertainty about inclusion.

**Figure 1:**
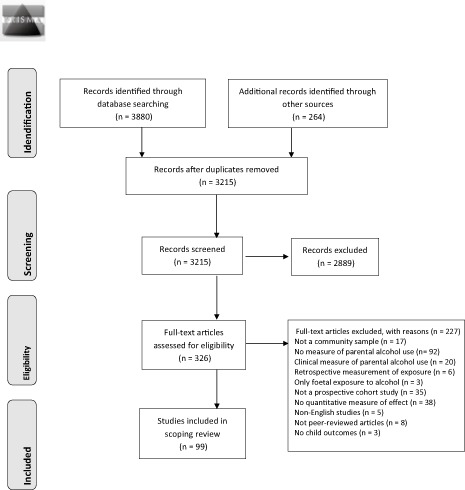
PRISMA 2009 flow diagram.

A summary of the selection process is illustrated in the PRISMA flowchart (Figure 1). Details of studies in this area not meeting selection criteria are provided in an online appendix (Appendix 1, available as a web‐based Supplement to this article) as an aid to further study. We followed PRISMA guidance on reporting but did not publish a protocol for this study, nor included it in a registry.

### Data analysis

Two authors (P. K. and L. F.) extracted relevant characteristics (participants, exposure, study design, outcomes) of all the included studies using a structured data collection tool. One author (I. R.) extracted additional study characteristics (type of analysis and study focus). This provided the basis of the systematic mapping of the included studies in terms of their characteristics and findings and the summary data presented here. Study characteristics were also analysed by cross‐tabulations and χ^2^ statistics. As this was a scoping review, we did not plan nor conduct assessment of risk of bias of the included studies, nor did we interrogate the strength of the evidence in relation to particular outcomes.

## Results

### The extent and range of studies

There were multiple study reports based on the same cohorts; altogether 60 distinct cohorts were identified. The characteristics of the literature comprising 99 studies eligible for inclusion are briefly summarised in Table 1. Parental drinking was measured in various ways. It varied with respect to child's age of exposure measurement (from infancy to young adulthood), measurement type (e.g. weekly number of drinks vs. dichotomous measure), specific behavioural focus (e.g. drinking frequency vs. problem drinking) and whether one or both parents' drinking behaviour was measured and analysed (see Table 1). Only a third of the studies (32%) had a primary focus on parental drinking as exposure measure. Also the child's age at outcome measurement varied considerably (from pre‐school age to mid adulthood). Several studies (*n* = 24) addressed associations between parental drinking and two or several types of outcomes (e.g. alcohol use and illicit drug use). Thus, the total number of reported associations between exposure and outcome category was 130. In a majority of these associations, the outcome measure was alcohol use or related harm (69%), whereas use of other substances (illicit drugs and tobacco) and various types of problems (e.g. psychological problems, externalising behaviour and crime) were outcome measures in 18% and 12% of the reported associations, respectively.

**Table 1:** Study characteristics

Characteristics	Distribution *n* (%)
Publication year (*n* = 99)	
1986–2000	27
2001–2007	30
2008–2014	42
Country (*n* = 99)	
The USA	51
The Netherlands	13
Australia	13
All other countries	22
Sample type (*n* = 99)	
Community sample	35
School students	34
Birth cohort	22
Twin study	4
Other	4
Sample size (*n* = 99)	
103–500	27
501–1000	28
1001–2000	15
2001+	29
Follow‐up years (*n* = 99)	
0.3–1.0	27
1.5–2.0	19
3.0–5.0	19
5.5–7.0	19
9.0–17.0	14
Exposure by who (*n* = 99)	
Separate for mother and father	28
Parents combined	43
Only mother	20
Only father	4
Other (e.g. ‘most important adult’)	4
Age of child at exposure measurement (*n* = 99)	
5.0–10.0 years	23
10.5–12.7 years	19
13.0–15.5 years	41
16.0–28.0 years	15
Exposure reported by (*n* = 99)	
Parent	67
Child	32
Outcome at age (*n* = 99)	
1.5–13.0 years	14
14.0–16.5 years	34
17.0–21.0 years	40
22.0–45.0 years	11
Study focus on parental drinking (*n* = 99)	
Primary focus	32
Among multiple factors examined in dedicated study	59
Covariate in analysis with other primary study focus	8
Outcome type (*n* = 130)	
Age of—or early initiation of alcohol use	11 (8.5)
Alcohol use, including heavy drinking	67 (51.5)
Alcohol related problems	12 (9.2)
Other substance use	24 (18.5)
Other outcomes	16 (12.3)
Findings (up to three outcomes) (*n* = 130)	
No association w/parental drinking	48 (36.9)
Some association with parental drinking (both or combined)	69 (53.1)
Some association with maternal drinking only	4 (3.1)
Some association with paternal drinking only	7 (5.4)
Only reversed association with parental drinking	0 (0)
Mixed findings for maternal and paternal drinking	2 (1.5)
Analysis type (*n* = 130)	
Bivariate	12 (9.2)
Multi‐variate	118 (90.8)

*Note*: For the first 10 study, characteristics *n* = 99 and percent corresponds closely to *n*. The final three study characteristics (outcome type, finding and analysis type) pertain to the total number of associations reported rather than individual studies (i.e. *n* = 130).

Table 2 presents study characteristics cross tabulated by the three categories of outcome measure (total *n* = 130). Studies examining outcome measures other than alcohol use or related problems were, on average, based on larger study samples and average age of measurement of child's exposure to parental drinking was lower.

**Table 2:** Distribution of study characteristics by outcome measure categories. *n*'s (percentage in parentheses). Total *n* = 130

Study characteristics	Alcohol use or related problems, *n* = 90 (%)	Other substance use or dependence, *n* = 24 (%)	Other behaviour problems, *n* = 16 (%)
Publication year			
1986–2000	32 (36)	4 (17)	3 (19)
2001–2007	26 (29)	9 (38)	4 (25)
2008–2014	32 (36)	11 (46)	9 (56)
Country			
The USA	49 (54)	7 (29)	13 (81)
The Netherlands	14 (16)	0 (0)	1 (6)
Australia	7 (8)	9 (38)	0 (0)
Other countries	20 (22)	8 (33)	2 (13)
Average sample size	1366	2262	4055
Average no of follow‐up years	3.8	7.0	3.3
Average age (years) at child's exposure	13.5	10.1	11.3
Average age (years) at child's outcome	17.5	17.1	16.5
Study focus on parental drinking			
Primary focus	29 (32)	4 (17)	6 (38)
Among multiple factors examined	56 (62)	15 (63)	9 (56)
Covariate in analysis with other focus	5 (6)	5 (21)	1 (6)

### Summary of included studies' findings

The association between parental drinking and the outcome variable was not statistically significant in a considerable proportion of the 130 reported associations (37%). The likelihood of finding an association with parental drinking (i.e. more parental drinking predicted increased risk of substance use or psycho‐social problem) was larger when this association was the primary study focus, when the exposure measure was obtained for both parents separately, when time to follow‐up exceeded 3 years and when the sample size exceeded 2000 respondents. Other study characteristics were not significantly associated with the likelihood of finding an association with parental drinking (Table 3).

**Table 3:** Distribution of study findings by study characteristics. *n*'s (percentage in parentheses). Total *n* = 130

Study characteristics	Reporting a statistically significant association between child outcome and parental drinking		χ^2^ Statistics
	Yes	No	
Publication year			χ = 3.03, *P* = 0.219
1986–2000	24 (62)	15 (39)	—
2001–2007	20 (51)	19 (49)	—
2008–2014	36 (69)	16 (31)	—
Exposure reported by			χ = 3.41, *P* = 0.065
Parent	56 (68)	27 (33)	—
Child	24 (51)	23 (49)	—
Sample size			χ = 5.57, *P* = 0.018
<2000	50 (55)	41 (45)	—
2000+	30 (77)	9 (23)	—
Number of follow‐up years			χ = 4.93, *P* = 0.026
<3 years	34 (53)	30 (47)	—
3+ years	46 (70)	20 (30)	—
Exposure measure obtained for whom			χ = 10.30, *P* = 0.036
Both parents separately	26 (76)	8 (24)	—
Both parents combined	29 (51)	28 (49)	—
Only mother	15 (65)	8 (35)	—
Only father	7 (87)	1 (13)	—
Other	3 (38)	5 (63)	—
Type of outcome measure			χ = 2.15, *P* = 0.341
Alcohol use or related problems	59 (66)	31 (34)	—
Other substance use	12 (50)	12 (50)	—
Other outcomes	9 (56)	7 (44)	—
Study focus on parental drinking			χ = 9.20, *P* = 0.010
Primary focus	31 (80)	8 (21)	—
Among multiple factors examined	45 (56)	35 (44)	—
Covariate in analysis with other focus	4 (36)	7 (64)	—

Of the 75 studies that analysed drinking behaviour as an outcome, 22 applied separate measures of mother's and father's drinking. Of these, 21 found an association of some kind. While approximately half of the studies (*n* = 11) found that both parents' drinking predicted drinking behaviour in offspring, the other half found that only one of the parents' drinking was associated with that of their offspring; either mother's drinking (*n* = 6) or father's drinking (*n* = 4). In 41 of the 75 studies, a combined measure of parental drinking was applied and 24 of these found an association between parental and offspring drinking behaviour, whereas no association was found in the other 17 studies.

Appendix 2 (available as a web‐based Supplement to this article) presents an overview of all the included studies and provides more detail about the main study findings from each study.

### Limitations and gaps in the literature

This literature has several limitations if one seeks to assess a possible causal role of parental drinking in children's adverse outcomes. First, the majority of studies were not primarily concerned with the association between parental drinking and subsequent outcomes in children. This reflects the limitations in the use of theoretically based analytical investigations of this issue, and hampers consideration of causal inference. Second, many studies had small samples, giving low statistical power to detect effects that are small or differential effects of the drinking of either parent. Third, exposure measures were often (44 of 99 studies) obtained in few and crude categories, which weakened the potential to address possible dose–response relationships. Fourth, in some studies, exposure was measured when children most likely had left home, and was therefore less likely to show a possible impact of parental drinking. Finally, in some studies (9% of reported associations), no adjustment for confounding factors was included in the analysis.

Few studies included in this review addressed outcomes other than alcohol or other substance use and related problems. This is noteworthy, as there is a substantial literature on the prevalence and elevated risk of psycho‐social problems in ‘children of alcoholics’. This literature demonstrates that parental alcoholism/alcohol abuse is associated with children's increased risk of various psychosocial problems, including conduct disorder; mood disorder and depressive symptoms; academic underachievement; low self‐esteem; emotional, physical and sexual abuse; and relational difficulties [15, 28, 29].

Although alcohol consumption is widespread globally, the literature in this area—like most other health and social areas—stems from a few high income countries. Only two studies were conducted in low and middle income countries: Brazil and Taiwan [30, 31]. Thus, there is meagre potential to appreciate possible cross‐cultural differences in the extent of alcohol related harm attributable to parental drinking.

## Discussion

This scoping review is—to our knowledge—the first comprehensive attempt to summarise the data available in cohort studies of parental drinking and adverse effects on their children. This study reveals that a large literature is available. In most studies, adolescent drinking behaviour was the outcome measure, and few studies have addressed adverse outcomes other than substance use. Parental drinking was found to be statistically significantly associated with a child harm outcome measure in almost two of every three published associations.

Two previous systematic reviews are, in part, thematically overlapping with this scoping review. One review [23] included 29 cohort studies of parental and adolescent drinking and 23 of these were among the 75 studies included in this scoping review. Ryan and co‐workers [23] found that in about two out of three studies, parental drinking was predictive of adolescent alcohol use, similar to the present findings. The other review [24] included 22 studies in which the exposure measure was substance abuse or illicit drug use and none were eligible for inclusion in our scoping review.

Notably, in about a third of the studies, there were no statistically significant associations, which may, in part, be ascribed to various study characteristics, including, for instance, parental drinking not being primary study focus and insufficient statistical power. Moreover, causal inferences are not warranted for child harms arising from parental drinking on the basis of the observed associations reported in this review. Although the prospective cohort study design and wide use of adjustment for covariates are needed in this regard, alternative explanations for study findings must also be considered. These explanations include spurious associations due to insufficient control for confounding factors, the effects of a range of biases and statistical artefacts in individual studies, and problems affecting the available literature as a whole, such as publication bias.

This review included only prospective cohort studies, which provide a stronger basis for assessment of causality in relation to the observed associations than other observational epidemiological designs. The exhaustive search for, and examination of, all published literature in this area is a clear strength of this study. The included studies varied greatly in definitions and measurements used for exposure and outcome variables and also in the approaches to analysis. Comparisons between the studies are therefore limited. All 99 studies were published in peer‐reviewed journals, which may be indicative of some study quality; however, we have not assessed risk of bias. Studies in languages other than English were not included, which may have biased the findings. Finally, publication bias should also be taken into account when interpreting these study findings [32, 33, 34]. Researchers may be less interested in publishing, or encounter difficulties in publishing null findings as journals may be less interested in them. This may have contributed to a distorted picture of the overall research evidence that exists, making studies reporting statistically significant associations more prominent than they should be. This risk exists for all studies of this type, and we note both the proportion of published studies that find no association and also the greater likelihood of statistically significant associations among the dedicated studies.

This review provides a basis for more analytic evaluations of the possible effects of parental drinking on the health and welfare of their children, and the mechanisms through which they occur. In particular, analytic reviews of possible impacts of parental drinking on that of their children seem feasible, as most of the existing cohort studies have addressed this association and further datasets are available. There is clearly a need for more cohort studies that specifically focus on parental drinking and outcomes in children, and, in particular, there is a need for such studies on adverse outcomes other than substance use. Preferably, such studies should be designed to account for the complexities and possible causal mechanisms involved in the relationships between parental drinking and child outcomes. Moreover, studies from countries outside North America, West Europe and Australia/New Zealand are particularly needed in order to obtain a better understanding of the possible harms from parental drinking across diverse socioeconomic and cultural settings.

## Supporting information


**Table S1.** Excluded studies by reason for exclusion.

**Table S2.** Overview of studies, grouped by type of outcome.
