# Supplementary material for: Parental drinking and adverse outcomes in children: A scoping review of cohort studies
Source: Drug Alcohol Rev. 2015 Aug 31;35(4):397–405. doi: 10.1111/dar.12319 (PMC4950034; doi:10.1111/dar.12319)
Supplement: Supplementary file 1 — Table S1. Excluded studies by reason for exclusion. [file DAR-35-397-s001.docx]

Appendix 1

Table S1: Excluded studies by reason for exclusion

| **Study ID** | **Not a general population sample** | **No measure of parental exposure** | **ICD/DSM measure of parental exposure** | **Retrospective measurement of exposure** | **Exposure to alcohol only pre-birth** | **Not a prospective cohort study** | **No quantitative measure of effect** | **Non-English language studies** | **Not peer-reviewed journals** | **No child outcomes** |
| --- | --- | --- | --- | --- | --- | --- | --- | --- | --- | --- |
| Abar 2008 [1] | . | ✓ | . | . | . | . | . | . | . | . |
| Abar 2009 [2] | . | . | . | . | . | . | . | . | ✓ | . |
| Adalbjarnardottir 2001 [3] | . | . | . | . | . | ✓ | . | . | . | . |
| Adams 2013 [4] | . | . | . | . | . | . | . | . | ✓ | . |
| Agrawal 2007 [5] | . | ✓ | . | . | . | . | . | . | . | . |
| Alati 2010 [6] | . | . | . | . | . | . | ✓ | . | . | . |
| Andrews 1997 [7] | . | . | . | . | . | ✓ | . | . | . | . |
| Ary 1988 [8] | . | ✓ | . | . | . | . | . | . | . | . |
| Ary 1999a [9] | . | ✓ | . | . | . | . | . | . | . | . |
| Ary 1999b [1]0 | . | ✓ | . | . | . | . | . | . | . | . |
| Aseltine 2000 [11] | . | . | . | . | . | . | ✓ | . | . | . |
| Bailey 2009 [12] | . | ✓ | . | . | . | . | . | . | . | . |
| Balsa 2012 [13] | . | . | . | . | . | ✓ | . | . | . | . |
| Barnes 1992 [14] | . | . | . | . | . | . | ✓ | . | . | . |
| Barnes 2000 [15] | . | . | . | . | . | . | ✓ | . | . | . |
| Baumrind 1985 [16] | . | ✓ | . | . | . | . | . | . | . | . |
| Bergmark 1999 [17] | . | ✓ | . | . | . | . | . | . | . | . |
| Beseler 2008 [18] | ✓ | . | . | . | . | . | . | . | . | . |
| Biddle 1980 [19] | . | . | . | . | . | ✓ | . | . | . | . |
| Bonomo 2004 [20] | . | . | . | . | . | ✓ | . | . | . | . |
| Branstorm 2007 [21] | . | . | . | . | . | ✓ | . | . | . | . |
| Bray 2001a [22] | . | ✓ | . | . | . | . | . | . | . | . |
| Bray 2001b [23]_ENREF_11 | . | ✓ | . | . | . | . | . | . | . | . |
| Brody 1993 [24] | . | ✓ | . | . | . | . | . | . | . | . |
| Brody 2001 [25]_ENREF_11 | . | ✓ | . | . | . | . | . | . | . | . |
| Brody 2000 [26] | . | ✓ | . | . | . | . | . | . | . | . |
| Brook 1986 [27] | . | . | . | . | . | ✓ | . | . | . | . |
| Brook 1995 [28] | . | ✓ | . | . | . | . | . | . | . | . |
| Brook 2001a [29] | . | . | . | . | . | . | ✓ | . | . | . |
| Brook 2001b [30] | . | . | . | . | . | ✓ | . | . | . | . |
| Brunswick 1983 [31] | . | ✓ | . | . | . | . | . | . | . | . |
| Cable 2008 [32] | . | . | . | . | . | ✓ | . | . | . | . |
| Casswell 1983 [33] | . | . | . | . | . | ✓ | . | . | . | . |
| Casswell 1985 [34] | . | . | . | . | . | ✓ | . | . | . | . |
| Casswell 1988 [35] | . | . | . | . | . | ✓ | . | . | . | . |
| Casswell 1991 [36] | . | . | . | . | . | . | ✓ | . | . | . |
| Chapple 2005 [37] | . | . | . | . | . | . | ✓ | . | . | . |
| Chilcoat 1996 [38]_ENREF_39 | . | ✓ | . | . | . | . | . | . | . | . |
| Christoffersen 2003 [39] | . | . | ✓ | . | . | . | . | . | . | . |
| Christoffersen 2008 [40] | ✓ | . | . | . | . | . | . | . | . | . |
| Chuang 2005 [41] | . | . | . | . | . | ✓ | . | . | . | . |
| Clark 2005 [42] | ✓ | . | . | . | . | . | . | . | . | . |
| Cleveland 2003 [43] | . | . | . | . | . | ✓ | . | . | . | . |
| Cleveland 2005 [44] | . | . | . | . | . | . | ✓ | . | . | . |
| Coffelt 2006 [45] | . | . | ✓ | . | . | . | . | . | . | . |
| Coker 2001 [46] | . | ✓ | . | . | . | . | . | . | . | . |
| Conger 1996 [47] | . | . | . | . | . | . | . | . | ✓ | . |
| Connolly 1992 [48] | . | ✓ | . | . | . | . | . | . | . | . |
| Connolly 1993 [49] | . | . | . | . | . | . | ✓ | . | . | . |
| Cranford 2010 [50] | ✓ | . | . | . | . | . | . | . | . | . |
| Crawford 2002 [51] | . | ✓ | . | . | . | . | . | . | . | . |
| Crawford 2008 [52] | . | ✓ | . | . | . | . | . | . | . | . |
| Crum 1996 [53] | . | . | . | ✓ | . | . | . | . | . | . |
| Cumes-Rayner 1992 [54] | ✓ | . | . | . | . | . | . | . | . | . |
| Curran 1996 [55] | ✓ | . | . | . | . | . | . | . | . | . |
| Danielsson 2011 [56] | . | ✓ | . | . | . | . | . | . | . | . |
| Dick 2007 [57] | . | . | . | . | . | . | ✓ | . | . | . |
| Dooley 2006 [58] | . | . | ✓ | . | . | . | . | . | . | . |
| Duncan 1995 [59] | . | ✓ | . | . | . | . | . | . | . | . |
| Duncan 1996 [60] | . | ✓ | . | . | . | . | . | . | . | . |
| Duncan 2006 [61] | . | . | . | . | . | . | ✓ | . | . | . |
| Duncan 1998 [62] | . | ✓ | . | . | . | . | . | . | . | . |
| East 2005 [63] | . | ✓ | . | . | . | . | . | . | . | . |
| Eisenberg 2008 [64] | . | ✓ | . | . | . | . | . | . | . | . |
| El sheikh 2001 [65] | . | . | . | . | . | ✓ | . | . | . | . |
| Elkington 2011 [66] | . | ✓ | . | . | . | . | . | . | . | . |
| Engels 2005 [67] | . | ✓ | . | . | . | . | . | . | . | . |
| Epstein 2013 [68] | . | . | . | . | . | . | ✓ | . | . | . |
| Felton 1999 [69] | . | . | . | . | . | . | ✓ | . | . | . |
| Fergusson 1998 [70] | . | ✓ | . | . | . | . | . | . | . | . |
| Fergusson 2002 [71] | ✓ | . | . | . | . | . | . | . | . | . |
| Flett 1987 [72] | . | ✓ | . | . | . | . | . | . | . | . |
| Flewelling 1990 [73] | . | ✓ | . | . | . | . | . | . | . | . |
| Gagnon 2011 [74] | . | . | . | . | . | . | . | ✓ | . | . |
| Galaif 2001 [75] | . | ✓ | . | . | . | . | . | . | . | . |
| Garg 2009 [76] | . | ✓ | . | . | . | . | . | . | . | . |
| Gerrard 1999 [77] | . | . | . | . | . | . | ✓ | . | . | . |
| Godleski 2013 [78] | . | . | . | . | . | . | . | . | ✓ | . |
| Griffin 2000 [79]_ENREF_74 | . | ✓ | . | . | . | . | . | . | . | . |
| Grijbovski 2002 [80] | . | . | . | . | ✓ | . | . | . | . | . |
| Guilamo-Ramos 2005 [81] | . | ✓ | . | . | . | . | . | . | . | . |
| Guo 2002 [82] | . | ✓ | . | . | . | . | . | . | . | . |
| Handley 2009 [83] | . | . | . | . | . | . | . | . | ✓ | . |
| Hanewinkel 2008 [84] | . | . | . | . | . | . | ✓ | . | . | . |
| Harburg 1982 [85] | . | . | . | . | . | . | ✓ | . | . | . |
| Harburg 1990a [86] | . | . | . | . | . | . | ✓ | . | . | . |
| Harburg 1990b [87] | . | . | . | . | . | . | ✓ | . | . | . |
| Harrington 2011 [88] | . | . | . | ✓ | . | . | . | . | . | . |
| Hayatbahksh 2008a [89]_ENREF_47 | . | . | . | . | . | . | ✓ | . | . | . |
| Hayatbakhsh 2006 [90]_ENREF_30_ENREF_48 | . | . | . | . | . | . | ✓ | . | . | . |
| Hayatbakhsh 2008b [91] | . | . | . | . | . | . | ✓ | . | . | . |
| Hayatbakhsh 2011a [92] | . | . | . | . | ✓ | . | . | . | . | . |
| Hayatbaksh 2011b [93] | . | . | . | . | . | . | ✓ | . | . | . |
| Heath 1999 [94] | . | ✓ | . | . | . | . | . | . | . | . |
| Hendershot 2005 [95] | . | . | . | . | . | ✓ | . | . | . | . |
| Hoffman 1993[ 96] | . | ✓ | . | . | . | . | . | . | . | . |
| Hops 1999 [97] | . | ✓ | . | . | . | . | . | . | . | . |
| Huizink 2009 [98] | . | . | . | . | . | . | ✓ | . | . | . |
| Husong 2005 [99] | . | . | ✓ | . | . | . | . | . | . | . |
| Huurre 2010 [100] | . | ✓ | . | . | . | . | . | . | . | . |
| Iacono 1999 [101] | . | . | ✓ | . | . | . | . | . | . | . |
| Irons 2012 [102] | . | . | ✓ | . | . | . | . | . | . | . |
| Johnson 1991 [103] | . | . | . | . | . | . | ✓ | . | . | . |
| Johnson 1995 [104] | . | . | ✓ | . | . | . | . | . | . | . |
| Jordan 2005 [105] | ✓ | . | . | . | . | . | . | . | . | . |
| Kaufman 2007 [106] | ✓ | . | . | . | . | . | . | . | . | . |
| Keller 2008 [107] | . | . | ✓ | . | . | . | . | . | . | . |
| Keller 2011 [108] | . | . | ✓ | . | . | . | . | . | . | . |
| Kim 2003 [109] | . | ✓ | . | . | . | . | . | . | . | . |
| Knappe 2009 [110] | . |  | ✓ | . | . | . | . | . | . | . |
| Komro 2007 [111] | . | ✓ | . | . | . | . | . | . | . | . |
| korhonen 2012 [112] | . | ✓ | . | . | . | . | . | . | . | . |
| Kosterman 2000 [113] | . | ✓ | . | . | . | . | . | . | . | . |
| Kuntsche 2009 [114] | . |  | . | . | . | . | ✓ | . | . | . |
| Lamis 2012 [115] | . | ✓ | . | . | . | . | . | . | . | . |
| Lau 1990 [116] | . | . | . | . | . | . | ✓ | . | . | . |
| Lee 2011 [117] | . | . | . | . | . | . | . | . | . | ✓ |
| Lee 2012 [118] | . | . | . | . | . | . | . | . | . | ✓ |
| Li 2000 [119] | . | ✓ | . | . | . | . | . | . | . | . |
| Li 2001 [120] | . | ✓ | . | . | . | . | . | . | . | . |
| Licanin 2006 [121] | . | ✓ | . | . | . | . | . | . | . | . |
| Livaudais 2007 [122] | . | ✓ | . | . | . | . | . | . | . | . |
| Lynne-landsman 2010 [123] | . | ✓ | . | . | . | . | . | . | . | . |
| Lynskey 1998 [124] | . | ✓ | . | . | . | . | . | . | . | . |
| Maggs 2008 [125] | . | ✓ | . | . | . | . | . | . | . | . |
| Malone 2002 [126] | . | . | ✓ | . | . | . | . | . | . | . |
| Marshal 2000 [127] | ✓ | . | . | . | . | . | . | . | . | . |
| Martino 2006 [128] | . | . | . | . | . | ✓ | . | . | . | . |
| McGue 1996 [129] | . | . | . | . | . | ✓ | . | . | . | . |
| Mclaughlin 1985 [130] | . | . | . | . | . | ✓ | . | . | . | . |
| McMorris 2002 [131[ | ✓ | . | . | . | . | . | . | . | . | . |
| McMorris 2011 [132] | . | ✓ | . | . | . | . | . | . | . | . |
| Merline 2008 [133] | . | . | . | ✓ | . | . | . | . | . | . |
| Mogro-Wilson 2008 [134] | . | ✓ | . | . | . | . | . | . | . | . |
| Morojele 2001 [135] | . | ✓ | . | . | . | . | . | . | . | . |
| Najman 2005 [136] | . | . | . | . | ✓ | . | . | . | . | . |
| Nash 2005 [137] | . | ✓ | . | . | . | . | . | . | . | . |
| Needle 1986 [138] | . | . | . | . | . | ✓ | . | . | . | . |
| Newcomb 1992 [139] | . | ✓ | . | . | . |  | . | . | . | . |
| Noal 2010 [140] | . | . | . | . | . | ✓ | . | . | . | . |
| Ohannessian 2012 [141] | . | . | ✓ | . | . | . | . | . | . | . |
| Ostaszewski 2006 [142] | . | ✓ | . | . | . | . | . | . | . | . |
| Oxford 2001 [143] | . | ✓ | . | . | . | . | . | . | . | . |
| Pagan 2006 [144] | . | ✓ | . | . | . | . | . | . | . | . |
| Pandina 1989 [145] | . | . | . | . | . | . | ✓ | . | . | . |
| Peck 2008 [146] | . | ✓ | . | . | . | . | . | . | . | . |
| Percy 2007 [147] | . | . | . | . | . | ✓ | . | . | . | . |
| Percy 2008 [148] | . | . | . | . | . | . | ✓ | . | . | . |
| Pitkanen 2008 [149] | . | . | . | ✓ | . | . | . | . | . | . |
| Poikolainen 2001 [150] | . | . | . | . | . | . | ✓ | . | . | . |
| Prior 1999 [151] | . | ✓ | . | . | . | . | . | . | . | . |
| Pulkkinen 1983 [152] | . | . | . | . | . | ✓ | . | . | . | . |
| Pulkkinen 1994 [153] | . | . | . | ✓ | . | . | . | . | . | . |
| Pulkkinen 2009 [154] | . | . | . | ✓ | . | . | . | . | . | . |
| Rantakallio 1983 [155] | . | ✓ | . | . | . | . | . | . | . | . |
| Rew 2011 [156] | . | ✓ | . | . | . | . | . | . | . | . |
| Rhee 2003 [157] | . | . | . | . | . | ✓ | . | . | . | . |
| Roche 2008 [158] | . | ✓ | . | . | . | . | . | . | . | . |
| Rodgers-Farmer 2000 [159] | . | ✓ | . | . | . | . | . | . | . | . |
| Roosa 1993 [160] | ✓ | . | . | . | . | . | . | . | . | . |
| Rose 1999 [161] | . | . | . | . | . | ✓ | . | . | . | . |
| Rossow 2013 [162] | . | . | . | . | . | . | ✓ | . | . | . |
| Schact 2009 [163] | ✓ | . | . | . | . | . | . | . | . | . |
| Schmid 2008 [164] | . | . | . | . | . | . | . | ✓ | . | . |
| Schmid 2010 [165] | ✓ | . | . | . | . | . | . | . | . | . |
| Scholes-Balog 2013 [166] | . | ✓ | . | . | . | . | . | . | . | . |
| Scholte 2008 [167] | . | . | . | . | . | ✓ | . | . | . | . |
| Seljamo 2006 [168] | . | . | . | . | . | ✓ | . | . | . | . |
| Sellers 1990 [169] | . | ✓ | . | . | . | . | . | . | . | . |
| Shucksmith 1997 [170] | . | ✓ | . | . | . | . | . | . | . | . |
| Siebenbruner 2006 [171] | . | ✓ | . | . | . | . | . | . | . | . |
| Sieving 2000 [172] | . | . | . | . | . | . | ✓ | . | . | . |
| Simons-Morton 2004 [173] | . | ✓ | . | . | . | . | . | . | . | . |
| Simons-Morton 2007 [174] | . | ✓ | . | . | . | . | . | . | . | . |
| Skeer 2009 [175] | . | ✓ | . | . | . | . | . | . | . | . |
| Sobeck 2000 [176] | . | ✓ | . | . | . | . | . | . | . | . |
| Sorensen 2011 [177] | . | . | ✓ | . | . | . | . | . | . | . |
| Sourander 2006 [178] | . | ✓ | . | . | . | . | . | . | . | . |
| Spein 2006 [179] | . | . | . | . | . | . | ✓ | . | . | . |
| Stanton 1992 [180] | . | ✓ | . | . | . | . | . | . | . | . |
| Stanton 2004 [181] | . | ✓ | . | . | . | . | . | . | . | . |
| Steele 1995 [182] | . | ✓ | . | . | . | . | . | . | . | . |
| Steinberg 1994 [183] | . | ✓ | . | . | . | . | . | . | . | . |
| Stewart 2002 [184] | . | ✓ | . | . | . | . | . | . | . | . |
| Stice 1998 [185] | . | . | ✓ | . | . | . | . | . | . | . |
| Stoolmiller 2012 [186] | . | . | . | . | . | ✓ | . | . | . | . |
| Suzuki 2002 [187] | . | . | . | . | . | . | . | ✓ | . | . |
| Suzuki 2005 [188] | . | . | . | . | . | . | . | ✓ | . | . |
| Suzuki 2007 [189] | . | . | . | . | . | . | . | ✓ | . | . |
| Thomas 2000 [190] | . | ✓ | . | . | . | . | . | . | . | . |
| Tildesley 2008 [191] | . | . | . | . | . | . | . | . | . | ✓ |
| Ting 2013 [192] | . | . | . | . | . | . | . | . | ✓ | . |
| Trim 2010 [193] | . | . | ✓ | . | . | . | . | . | . | . |
| Turner 2000 [194] | . | . | ✓ | . | . | . | . | . | . | . |
| Urberg 2005 [195] | ✓ | . | . | . | . | . | . | . | . | . |
| Van der Vorst 2006 [196] | . | ✓ | . | . | . | . | . | . | . | . |
| Van der Vorst 2007 [197] | . | ✓ | . | . | . | . | . | . | . | . |
| Van der Vorst 2010b [198] | . | ✓ | . | . | . | . | . | . | . | . |
| Van der Zwaluw 2008 [199] | . | . | ✓ | . | . | . | . | . | . | . |
| Van Ryzin 2012 [200] | . | ✓ | . | . | . | . | . | . | . | . |
| Van Zundert 2006 [201] | . | . | . | . | . | ✓ | . | . | . | . |
| Verges 2012 [202] | . | . | . | . | . | . | . | . | ✓ | . |
| Vicary 1986 [203] | . | ✓ | . | . | . | . | . | . | . | . |
| Visser 2013 [204] | . | . | . | . | . | . | ✓ | . | . | . |
| Von Borczyskowski 2011 [205] | . | . | ✓ | . | . | . | . | . | . | . |
| Walls 2009 [206] | . | ✓ | . | . | . | . | . | . | . | . |
| Warner 2003 [207] | . | . | ✓ | . | . | . | . | . | . | . |
| Webb 1995 [208] | . | . | . | . | . | ✓ | . | . | . | . |
| Webb 2002 [209] | . | ✓ | . | . | . | . | . | . | . | . |
| Weil 1994 [210] | . | . | . | . | . | . | ✓ | . | . | . |
| Wells 2004 [211] | . | . | . | . | . | . | ✓ | . | . | . |
| Westling 2008 [212] | . | ✓ | . | . | . | . | . | . | . | . |
| Widom 2007 [213] | ✓ | . | . | . | . | . | . | . | . | . |
| Williams 2000 [214] | . | . | . | . | . | . | . | . | ✓ | . |
| Wills 1996 [215] | . | . | . | . | . | . | ✓ | . | . | . |
| Windle 1996 [216] | . | ✓ | . | . | . | . | . | . | . | . |
| Windle 1999 [217] | . | . | . | . | . | . | ✓ | . | . | . |
| Winqvist 2007 [218] | . | . | ✓ | . | . | . | . | . | . | . |
| Wong 2006 [219] | ✓ | . | . | . | . | . | . | . | . | . |
| Wood 2004 [220] | . | ✓ | . | . | . | . | . | . | . | . |
| Wu 2006 [221] | . | ✓ | . | . | . | . | . | . | . | . |
| Yang 2012 [222] | . | . | . | . | . | . | ✓ | . | . | . |
| Yen 2007 [223] | . | . | . | . | . | ✓ | . | . | . | . |
| Yu 2003 [224] | . | . | . | . | . | ✓ | . | . | . | . |
| Zhang 2012 [225] | . | . | . | . | . | ✓ | . | . | . | . |
| Zhang 1999 [226] | . | . | . | . | . | ✓ | . | . | . | . |
| Zhang 1997 [227] | . | . | . | . | . | ✓ | . | . | . | . |
| **TOTAL** | **17** | **92** | **20** | **6** | **3** | **35** | **38** | **5** | **8** | **3** |

**REFERENCES**

1. Abar C, Turrisi R. How important are parents during the college years? A longitudinal perspective of indirect influences parents yield on their college teens' alcohol use. Addict Beha 2008;33:1360-8.

2. Abar C, Fernandez A, Wood M. The impact of alcohol related communications between parents and teens on college drinking: A latent class analysis. Alcohol Clin Exp Res 2009;33:174A.

3. Adalbjarnardottir S, Rafnsson FD. Perceived control in adolescent substance use: Concurrent and longitudinal analyses. Psychol Addict Behav 2001;15:25-32.

4. Adams LJ, Reich RR, Goldman MS. Parental expectancies and alcohol expectancy development in preadolescents: A longitudinal approach. Alcohol Clin Exp Res 2013;37:216A.

5. Agrawal A, Lynskey MT, Bucholz KK, Madden PAF, Heath AC. Correlates of cannabis initiation in a longitudinal sample of young women: The importance of peer influences. Prev Med 2007;45:31-4.

6. Alati R, Maloney E, Hutchinson DM, Najman JM, Mattick RP, Bor W, et al. Do maternal parenting practices predict problematic patterns of adolescent alcohol consumption? Addiction (Abingdon, England). 2010;105:872-80.

7. Andrews JA, Hops H, Duncan SC. Adolescent modeling of parent substance use: The moderating effect of the relationship with the parent. J Fam Psychol 1997;11:259-70.

8. Ary D, Biglan A. Longitudinal changes in adolescent cigarette smoking behavior: Onset and cessation. J Behavl Med 1988;11:361-82.

9. Ary DV, Duncan TE, Duncan SC, Hops H. Adolescent problem behavior: the influence of parents and peers. Behav Rese Ther 1999;37:217-30.

10. Ary DV, Duncan TE, Biglan A, Metzler CW, Noell JW, Smolkowski K. Development of adolescent problem behavior. J Abnorm Child Psychol 1999;27:141-50.

11. Aseltine RH, Gore SL. The variable effects of stress on alcohol use from adolescence to early adulthood. Subst Use Misuse. 2000;35:643-68.

12. Bailey JA, Hill KG, Oesterle S, Hawkins JD. Parenting practices and problem behavior across three generations: monitoring, harsh discipline, and drug use in the intergenerational transmission of externalizing behavior. Dev Psychol 2009;45:1214-26

13. Balsa AI, Homer JF, French MT. The health effects of parental problem drinking on adult children. J Ment Health Policy Econ 2009;12:55-66.

14. Barnes BM, Farrell MP. Parental support and control as predictors of adolescent drinking, delinquency, and related problem behaviors. Journal of Marriage and Family. 1992;54:763-72.

15. Barnes BM, Reifman AS, Farrell MP, Dintcheff BA. The effects of parenting on the development of adolescent alcohol misuse: a six-wave latent growth model. Journal of Marriage and Family 2000;62:175-86.

16. Baumrind D. Familial antecedents of adolescent drug use: A developmental perspective. In: Jones CL, Battjes RJ, editors. Etiology of drug abuse: Implications for prevention. Washington, DC: National Institute on Drug Abuse; 1985. p. 13-44.

17. Bergmark KH, Andersson T. The development of advanced drinking habits in adolescence--a longitudinal study. Subst Use Misuse 1999;3:171-94.

18. Beseler CL, Aharonovich E, Keyes KM, Hasin DS. Adult transition from at-risk drinking to alcohol dependence: the relationship of family history and drinking motives. Alcohol Clin Exp Res 2008;32:607-16.

19. Biddle BJ, Bank BJ, Marlin MM. Parental and peer influence on adolescents. Social Forces 1980;58:1057-79.

20. Bonomo YA, Bowes G, Coffey C, Carlin JB, Patton GC. Teenage drinking and the onset of alcohol dependence: a cohort study over seven years. Addiction 2004;99:1520-8.

21. Branstrom R, Sjostrom E, Andreasson S. Individual, group and community risk and protective factors for alcohol and drug use among Swedish adolescents. Eur J Public Health 2008;18:12-8.

22. Bray JH, Adams GJ, Getz JG, Baer PE. Developmental, family, and ethnic influences on adolescent alcohol usage: a growth curve approach. J Fam Psychol 2001;15:301-14.

23. Bray JH, Adams GJ, Getz JG, Stovall T. Interactive effects of individuation, family factors, and stress on adolescent alcohol use. Am J Orthopsychiatry 2001;71:436-49

24. Brody GH, Forehand R. Prospective associations among family form, family processes, and adolescents alcohol and drug-use. Behav Res Ther 1993;31:587-93

25. Brody GH, Ge X. Linking parenting processes and self-regulation to psychological functioning and alcohol use during early adolescence. J Fam Psychol 2001;15:82-94.

26. Brody GH, Ge X, Katz J, Arias I. A longitudinal analysis of internalization of parental alcohol-use norms and adolescent alcohol use. Applied Developmental Science. 2000;4:71-9.

27. Brook JS, Whiteman M, Gordon AS, Cohen P. Some models and mechanisms for explaining the impact of maternal and adolescent characteristics on adolescent stage of drug use. Dev Psychol 1986;22:460-7.

28. Brook JS, Whiteman M, Gordon AS, Brook DW. Father's influence on his daughter's marijuana use viewed in a mother and peer context. Adv Alcohol Subst Abuse 1985;4:165-90.

29. Brook JS, Brook DW, Arencibia-Mireles O, Richter L, Whiteman M. Risk factors for adolescent marijuana use across cultures and across time. J Genet Psychol 2001;162:357-74.

30. Brook JS, Brook DW, De La Rosa M, Whiteman M, Johnson E, Montoya I. Adolescent illegal drug use: the impact of personality, family, and environmental factors. J Behav Med 2001;24:183-203.

31. Brunswick AF, Messeri P. Causal factors in onset of adolescents' cigarette smoking: a prospective study of urban black youth. Adv Alcohol Subst Abuse 1983;3:35-52.

32. Cable N, Sacker A. Typologies of alcohol consumption in adolescence: Predictors and adult outcomes. Alcohol Alcohol 2008;43:81-90.

33. Casswell S, Gilmore L, Silva P, Brasch P. Early experiences with alcohol: a survey of an eight and nine year old sample. N Z Med J 1983;96:1001-3.

34. Casswell S, Brasch P, Gilmore L, Silva P. Children's attitudes to alcohol and awareness of alcohol-related problems. Br J Addict 1985;80:191-4.

35. Casswell S, Gilmore LL, Silva P, Brasch P. What children know about alcohol and how they know it. Br J Addict 1988;83:223-7.

36. Casswell S, Stewart J, Connolly G, Silva P. A longitudinal study of New Zealand children's experience with alcohol. Br J Addict 1991;86:277-85.

37. Chapple CJ, Trina LH, Whiteford SW. The direct and indirect effects of parental bonds, parental drug use, and self-control on adolescent substance use. Journal of Child and Adolescent Substance Abuse. 2005;4:17-38.

38. Chilcoat HD, Breslau N. Alcohol disorders in young adulthood: Effects of transitions into adult roles. J Health Soc Behav 1996;37:339-49.

39. Christoffersen MN, Soothill K. The long-term consequences of parental alcohol abuse: a cohort study of children in Denmark. J Subst Abuse Treat 2003;25:107-16.

40. Christoffersen MN, Soothill K, Francis B. Risk factors for a first-time drink-driving conviction among young men: A birth cohort study of all men born in Denmark in 1966. J Subst Abuse Treat 2008;34:415-25.

41. Chuang YC, Ennett ST, Bauman KE, Foshee VA. Neighborhood influences on adolescent cigarette and alcohol use: mediating effects through parent and peer behaviors. J Health Soc Behav 2005;46:187-204.

42. Clark DB, Thatcher DL, Maisto SA. Supervisory neglect and adolescent alcohol use disorders: Effects on AUD onset and treatment outcome. Addict Behav 2005;30:1737-50.

43. Cleveland HH, Wiebe RP. The moderation of genetic and shared-environmental influences on adolescent drinking by levels of parental drinking. J Stud Alcohol 2003;64:182-94.

44. Cleveland MJ, Gibbons FX, Gerrard M, Pomery EA, Brody GH. The impact of parenting on risk cognitions and risk behavior: A study of mediation and moderation in a panel of African American adolescents. Child Dev 2005;76:900-16

45. Coffelt NL, Forehand R, Olson AL, Jones DJ, Gaffney CA, Zens MS. A longitudinal examination of the link between parent alcohol problems and youth drinking: The moderating roles of parent and child gender. Addict Behav 2006;31:593-605

46. Coker JK, Borders LD. An analysis of environmental and social factors affecting adolescent problem drinking. Journal of Counseling & Development. 2001;79:200-8

47. Conger RD, Rueter MA. Siblings, parents, and peers: A longitudinal study of social influences in adolescent risk for alcohol use and abuse. Sibling relationships: Their causes and consequences. Westport, CT: Ablex Publishing; US; 1996. p. 1-30.

48. Connolly GM, Casswell S, Stewart J, Silva PA. Drinking context and other influences on the drinking of 15-year-old New Zealanders. Br J Addict 1992;87:1029-36

49. Connolly GM, Casswell S, Stewart J, Silva PA, O'Brien MK. The effect of parents' alcohol problems on children's behaviour as reported by parents and by teachers. Addiction 1993;88:1383-90

50. Cranford JA, Zucker RA, Jester JM, Puttler LI, Fitzgerald HE. Parental Alcohol Involvement and Adolescent Alcohol Expectancies Predict Alcohol Involvement in Male Adolescents. Psychol Addict Behav 2010;24:386-96

51. Crawford LA, Novak KB. Parental and peer influences on adolescent drinking: The relative impact of attachment and opportunity. Journal of Child & Adolescent Substance Abuse. 2002;12:1-26

52. Crawford LA, Novak KB. Parent–child relations and peer associations as mediators of the family structure–substance use relationship. Journal of Family Issues. 2008;29:155-84

53. Crum RM, Harris EL. Risk of alcoholism and parental history: Gender differences and a possible reporting bias. Genet Epidemiol 1996;13:329-41.

54. Cumes-Rayner DP, Lucke JC, Singh B, Adler B, Lewin T, Dunne M, et al. A high-risk community study of paternal alcohol consumption and adolescents' psychosocial characteristics. J Stud Alcohol 1992;53:626-35.

55. Curran PJ, Chassin L. A longitudinal study of parenting as a protective factor for children of alcoholics. J Stud Alcohol 1996;57:305-13.

56. Danielsson AK, Romelsjo A, Tengstrom A. Heavy episodic drinking in early adolescence: gender-specific risk and protective factors. Subst Use Misuse 2011;46:633-43

57. Dick DM, Pagan JL, Viken R, Purcell S, Kaprio J, Pulkkinen L, et al. Changing environmental influences on substance use across development. Twin Res Hum Genet 2007;10:315-26.

58. Dooley D, Prause J. Predictors of early alcohol drinking onset. Journal of Child & Adolescent Substance Abuse. 2007;16:1-29.

59. Duncan TE, Duncan SC. Modeling the processes of development via latent variable growth curve methodology. Structural Equation Modeling-a Multidisciplinary Journal. 1995;2:187-213.

60. Duncan SC, Duncan TE, Hops H. Analysis of longitudinal data within accelerated longitudinal designs. Psychol Method 1996;1:236-48.

61. Duncan SC, Duncan TE, Strycker LA. Alcohol use from ages 9 to 16: A cohort-sequential latent growth model. Drug Alcohol Depend 2006;81:71-81.

62. Duncan SC, Duncan TE, Biglan A, Ary D. Contributions of the social context to the development of adolescent substance use: a multivariate latent growth modeling approach. Drug Alcohol Depend 1998;50:57-71.

63. East PL, Khoo ST. Longitudinal pathways linking family factors and sibling relationship qualities to adolescent substance use and sexual risk behaviors. J Fam Psychol 2005;19:571-80

64. Eisenberg ME, Neumark-Sztainer D, Fulkerson JA, Story M. Family meals and substance use: is there a long-term protective association? J Adolesc Health. 2008;43:151-6

65. El-Sheikh M, Flanagan E. Parental problem drinking and children's adjustment: family conflict and parental depression as mediators and moderators of risk. J Abnorm Child Psychol 2001;29:417-32

66. Elkington KS, Bauermeister JA, Zimmerman MA. Do parents and peers matter? A prospective socio-ecological examination of substance use and sexual risk among African American youth. J Adolesc 2011;34:1035-47

67. Engels RCME, Vermulst AA, Dubas JS, Bot SM, Gerris J. Long-term effects of family functioning and child characteristics on problem drinking in young adulthood. Eur Addict Res 2005;11:32-7

68. Epstein M, Hill KG, Bailey JA, Hawkins JD. The effect of general and drug-specific family environments on comorbid and drug-specific problem behavior: a longitudinal examination. Dev Psychol. 2013;49:1151-64

69. Felton G, Parsons MA, Ward DS, Pate RR, Saunders RP, Dowda M, et al. Tracking of avoidance of alcohol use and smoking behavior in a fifth grade cohort over three years. Public Health Nurs 1999;16:32-40

70. Fergusson DM, Horwood LJ. Early conduct problems and later life opportunities. J Child Psychol Psychiatry 1998;39:1097-108

71. Fergusson DM, Swain-Campbell N, Horwood LJ. Outcomes of leaving school without formal educational qualifications. New Zealand Journal of Educational Studies 2002;37:39-55

72. Flett R, Casswell S, Brasch P, Silva P. Alcohol knowledge and experience in children aged 9 and 11. N Z Med J. 1987;100:747-9

73. Flewelling RL, Bauman KE. Family Structure as a Predictor of Initial Substance Use and Sexual Intercourse in Early Adolescence. Journal of Marriage and Family. 1990;52:171-81.

74. Gagnon V, Fallu J-S, Briere FN, Janosz M. Initiation of Ecstasy Use in Quebec Senior High School Adolescents: Distal and Proximal Predictors. Can J Psychiatry 2011;56:62-70.

75. Galaif ER, Stein JA, Newcomb MD, Bernstein DP. Gender differences in the prediction of problem alcohol use in adulthood: exploring the influence of family factors and childhood maltreatment. J Stud Alcohol. 2001;62:486-93.

76. Garg A, Chavan BS, Singh GP, Bansal E. Patterns of alcohol consumption in medical students. J Indian Med Assoc 2009;107:151-5.

77. Gerrard M, Gibbons FX, Zhao L, Russell DW, Reis-Bergan M. The effect of peers' alcohol consumption on parental influence: a cognitive mediational model J Stud Alcohol Suppl 1999;13:32-44.

78. Godleski SA, Eiden RD, Colder C, Leonard KE. The influence of parent alcohol use and anger on early childhood aggression. Alcohol Clin Exp Res 2013;37:71A.

79. Griffin KW, Botvin GJ, Epstein JA, Doyle MM, Diaz T. Psychosocial and behavioral factors in early adolescence as predictors of heavy drinking among high school seniors. J Stud Alcohol 2000;61:603-6.

80. Grjibovski A, Bygren LO, Svartbo B. Socio-demographic determinants of poor infant outcome in north-west Russia. Paediatr Perinat Epidemiol 2002;16:255-62.

81. Guilamo-Ramos V, Jaccard J, Turrisi R, Johansson M. Parental and school correlates of binge drinking among middle school students. Am J Public Health 2005;95:894-9.

82. Guo J, Hawkins JD, Hill KG, Abbott RD. Childhood and adolescent predictors of alcohol abuse and dependence in young adulthood. J Stud Alcohol 2001;62:754-62.

83. Handley ED, Chassin L. The intergenerational transmission of alcohol expectancies in a high-risk sample. Alcohol Clin Exp Res 2009;33:240A.

84. Hanewinkel R, Morgenstern M, Tanski SE, Sargent JD. Longitudinal study of parental movie restriction on teen smoking and drinking in Germany. Addiction 2008;103:1722-30.

85. Harburg E, Davis DR, Caplan R. Parent and offspring alcohol use; imitative and aversive transmission. J Stud Alcohol 1982;43:497-516.

86. Harburg E, DiFranceisco W, Webster DW, Gleiberman L, Schork A. Familial transmission of alcohol use: II. Imitation of and aversion to parent drinking (1960) by adult offspring (1977)--Tecumseh, Michigan. J Stud Alcohol 1990;51:245-56.

87. Harburg E, Gleiberman L, DiFranceisco W, Schork A, Weissfeld L. Familial transmission of alcohol use, III. Impact of imitation/non-imitation of parent alcohol use (1960) on the sensible/problem drinking of their offspring (1977). Br J Addict 1990;85:1141-55

88. Harrington M, Robinson J, Bolton SL, Sareen J, Bolton J. A longitudinal study of risk factors for incident drug use in adults: findings from a representative sample of the US population. Can J Psychiatry 2011;56:686-95.

89. Hayatbakhsh MR, McGee TR, Bor W, Najman JM, Jamrozik K, Mamun AA. Child and adolescent externalizing behavior and cannabis use disorders in early adulthood: an Australian prospective birth cohort study. Addict Behav 2008;33:422-38.

90. Hayatbakhsh MR, Najman JM, Jamrozik K, Mamun AA, Williams GM, Alati R. Changes in maternal marital status are associated with young adults' cannabis use: evidence from a 21-year follow-up of a birth cohort. Int J Epidemiol. 2006;35:673-9.

91. Hayatbakhsh MR, Najman JM, Jamrozik K, Al Mamun A, Bor W, Alati R. Adolescent problem behaviours predicting DSM-IV diagnoses of multiple substance use disorder. Findings of a prospective birth cohort study. Soc Psychiatry Psychiatr Epidemiol 2008;43:356-63

92. Hayatbakhsh MR, Najman JM, Khatun M, Al Mamun A, Bor W, Clavarino A. A longitudinal study of child mental health and problem behaviours at 14years of age following unplanned pregnancy. Psychiatry Res 2011;185:200-4

93. Hayatbakhsh MR, Najman JM, Bor W, Clavarino A, Alati R. School performance and alcohol use problems in early adulthood: A longitudinal study. Alcohol 2011;45:701-9

94. Heath AC, Madden PAF, Grant JD, McLaughlin TL, Todorov AA, Bucholz KK. Resiliency factors protecting against teenage alcohol use and smoking: Influences of religion, religious involvement and values, and ethnicity in the Missouri adolescent female twin study. Twin Res 1999;2:145-55

95. Hendershot CS, MacPherson L, Myers MG, Carr LG, Wall TL. Psychosocial, cultural and genetic influences on alcohol use in Asian American youth. J Stud Alcohol 2005;66:185-95

96. Hoffmann JP. Exploring the direct and indirect family effects on adolescent drug use. Journal of Drug Issues 1993;23:535-57

97. Hops H, Davis B, Lewin LM. The development of alcohol and other substance use: a gender study of family and peer context. J Stud Alcohol Suppl 1999;13:22-31.

98. Huizink AC, Greaves-Lord K, Oldehinkel AJ, Ormel J, Verhulst FC. Hypothalamic-pituitary-adrenal axis and smoking and drinking onset among adolescents: The longitudinal cohort TRacking Adolescents' Individual Lives Survey (TRAILS). Addiction. 2009;104:1927-36.

99. Hussong AM, Zucker RA, Wong MM, Fitzgerald HE, Puttler LI. Social competence in children of alcoholic parents over time. Dev Psychol. 2005;41:747-59.

100. Huurre T, Lintonen T, Kaprio J, Pelkonen M, Marttunen M, Aro H. Adolescent risk factors for excessive alcohol use at age 32 years. A 16-year prospective follow-up study. Soc Psychiatry Psychiatr Epidemiol 2010;45:125-34.

101. Iacono WG, Carlson SR, Taylor J, Elkins IJ, McGue M. Behavioral disinhibition and the development of substance-use disorders: findings from the Minnesota Twin Family Study. Dev Psychopathol 1999;11:869-900.

102. Irons DE, Iacono WG, Oetting WS, McGue M. Developmental trajectory and environmental moderation of the effect of ALDH2 polymorphism on alcohol use. Alcohol Clin Exp Res 2012;36:1882-91.

103. Johnson V, Pandina RJ. Effects of the family environment on adolescent substance use, delinquency, and coping styles. Am J Drug Alcohol Abuse 1991;17:71-88.

104. Johnson V. The relationship between parent and offspring comorbid disorders. J Subst Abuse 1995;7267-80.

105. Jordan LC, Lewis ML. Paternal relationship quality as a protective factor: preventing alcohol use among African American adolescents. Journal of Black Psychology. 2005;31:152-71.

106. Kaufman J, Yang BZ, Douglas-Palumberi H, Crouse-Artus M, Lipschitz D, Krystal JH, et al. Genetic and environmental predictors of early alcohol use. Biol Psychiatry 2007;61:1228-34.

107. Keller PS, Cummings EM, Davies PT, Mitchell PM. Longitudinal relations between parental drinking problems, family functioning, and child adjustment. Dev Psychopathol 2008;20:195-212.

108. Keller PS, Gilbert LR, Koss KJ, Cummings EM, Davies PT. Parental problem drinking, marital aggression, and child emotional insecurity: a longitudinal investigation. J Stud Alcohol Drugs 2011;72:711-22.

109. Kim KJ, Conger RD, Elder GH, Jr., Lorenz FO. Reciprocal influences between stressful life events and adolescent internalizing and externalizing problems. Child Dev 2003;74:127-43.

110. Knappe S, Lieb R, Beesdo K, Fehm L, Low NC, Gloster AT, et al. The role of parental psychopathology and family environment for social phobia in the first three decades of life. Depress Anxiety. 2009;26:363-70.

111. Komro KA, Maldonado-Molina MM, Tobler AL, Bonds JR, Muller KE. Effects of home access and availability of alcohol on young adolescents' alcohol use. Addiction 2007;102:1597-608.

112. Korhonen T, Latvala A, Dick DM, Pulkkinen L, Rose RJ, Kaprio J, et al. Genetic and environmental influences underlying externalizing behaviors, cigarette smoking and illicit drug use across adolescence. Behav Genet 2012;42:614-25.

113. Kosterman R, Hawkins JD, Guo J, Catalano RF, Abbott RD. The dynamics of alcohol and marijuana initiation: patterns and predictors of first use in adolescence. Am J Public Health 2000;90:360-6.

114. Kuntsche E, Van Der Vorst H, Engels R. The earlier the more? Differences in the links between age at first drink and adolescent alcohol use and related problems according to quality of parent-child relationships. J Stud Alcohol Drugs. 2009;70:346-54.

115. Lamis DA, Malone PS, Lansford JE, Lochman JE. Maternal depressive symptoms as a predictor of alcohol use onset and heavy episodic drinking in youths. J Consult Clin Psychol. 2012;80:887-96.

116. Lau RR, Quadrel MJ, Hartman KA. Development and change of young adults' preventive health beliefs and behavior: influence from parents and peers. J Health Soc Behav 1990;31:240-59.

117. Lee SJ, Altschul I, Shair SR, Taylor CA. Hispanic fathers and risk for maltreatment in father-involved families of young children. J Soc Social Work Res. 2011;2:125-42.

118. Lee SJ, Taylor CA, Bellamy JL. Paternal depression and risk for child neglect in father-involved families of young children. Child Abuse Negl 2012;36:461-9.

119. Li X, Stanton B, Feigelman S. Impact of perceived parental monitoring on adolescent risk behavior over 4 years. J Adolesc Health 2000;27:49-56.

120. Li F, Duncan TE, Hops H. Examining developmental trajectories in adolescent alcohol use using piecewise growth mixture modeling analysis. J Stud Alcohol 2001;62:199-210.

121. Licanin I, Redzic A, Ibrahimagic E. Alcoholism during adolescence. Med Arh 2006;60:225-9.

122. Livaudais JC, Napoles-Springer A, Stewart S, Kaplan CP. Understanding latino adolescent risk behaviors: Parental and peer influences. Ethn Dis 2007;172:298-304.

123. Lynne-Landsman SD, Graber JA, Andrews JA. Do trajectories of household risk in childhood moderate pubertal timing effects on substance initiation in middle school? Dev Psychol 2010;46:853-68.

124. Lynskey MT, Fergusson DM, Horwood LJ. The origins of the correlations between tobacco, alcohol, and cannabis use during adolescence. J Child Psychol Psychiatry 1998;39:995-1005.

125. Maggs JL, Patrick ME, Feinstein L. Childhood and adolescent predictors of alcohol use and problems in adolescence and adulthood in the National Child Development Study. Addiction 2008;103:7-22.

126. Malone SM, Iacono WG, McGue M. Drinks of the father: Father's maximum number of drinks consumed predicts externalizing disorders, substance use, and substance use disorders in preadolescent and adolescent offspring. Alcohol Clin Exp Res 2002;26:1823-32.

127. Marshal MP, Chassin L. Peer Influence on Adolescent Alcohol Use: The Moderating Role of Parental Support and Discipline. Applied Developmental Science 2000;4:80-8.

128. Martino SC, Collins RL, Ellickson PL, Schell TL, McCaffrey D. Socio-environmental influences on adolescents' alcohol outcome expectancies: a prospective analysis. Addiction 2006;101:971-83.

129. McGue M, Sharma A, Benson P. Parent and sibling influences on adolescent alcohol use and misuse: Evidence from a US adoption cohort. J Stud Alcohol 1996;57:8-18.

130. McLaughlin RJ, Baer PE, Burnside MA, Pokorny AD. Psychosocial correlates of alcohol use at two age levels during adolescence. J Stud Alcohol 1985;46:212-8.

131. McMorris BJ, Tyler KA, Whitbeck LB, Hoyt DR. Familial and "on-the-street" risk factors associated with alcohol use among homeless and runaway adolescents. J Stud Alcohol 2002;63:34-43.

132. McMorris BJ, Catalano RF, Kim MJ, Toumbourou JW, Hemphill SA. Influence of family factors and supervised alcohol use on adolescent alcohol use and harms: similarities between youth in different alcohol policy contexts. J Stud Alcohol Drugs 2011;72:418-28.

133. Merline A, Jager J, Schulenberg JE. Adolescent risk factors for adult alcohol use and abuse: stability and change of predictive value across early and middle adulthood. Addiction 2008;103:84-99.

134. Mogro-Wilson C. The influence of parental warmth and control on Latino adolescent alcohol use. Hispanic Journal of Behavioral Sciences. 2008;30:89-105.

135. Morojele NK, Brook JS. Adolescent precursors of intensity of marijuana and other illicit drug use among adult initiators. J Genet Psychol 2001;162:430-50

136. Najman JM, Hallam D, Bor WB, O'Callaghan M, Williams GM, Shuttlewood G. Predictors of depression in very young children--a prospective study. Soc Psychiatry Psychiatr Epidemiol 2005;40:367-74.

137. Nash SG, McQueen A, Bray JH. Pathways to adolescent alcohol use: Family environment, peer influence, and parental expectations. J Adolesc Health. 2005;37:19-28.

138. Needle R, McCubbin H, Wilson M, Reineck R, Lazar A, Mederer H. Interpersonal influences in adolescent drug use--the role of older siblings, parents, and peers. Int J Addict 1986;21:739-66.

139. Newcomb MD, Felix-Ortiz M. Multiple protective and risk factors for drug use and abuse: cross-sectional and prospective findings. J Pers Soc Psychol. 1992;63:280-96.

140. Noal RB, Menezes AMB, Araujo CL, Hallal PC. [Experimental use of alcohol in early adolescence: The 11-year follow-up of the 1993 Pelotas (Brazil) birth cohort study]. Cad Saude Publica. 2010;26:1937-44.

141. Ohannessian CM. Parental problem drinking and adolescent psychosocial adjustment: the mediating role of adolescent–parent communication. Journal of Research on Adolescence. 2012;22:498-511.

142. Ostaszewski K, Zimmerman MA. The effects of cumulative risks and promotive factors on urban adolescent alcohol and other drug use: A longitudinal study of resiliency. Am J Community Psychol 2006;38:237-49.

143. Oxford ML, Harachi TW, Catalano RF, Abbott RD. Preadolescent predictors of substance initiation: a test of both the direct and mediated effect of family social control factors on deviant peer associations and substance initiation. Am J Drug Alcohol Abuse. 2001;27:599-616.

144. Pagan JL, Rose RJ, Viken RJ, Pulkkinen L, Kaprio J, Dick DM. Genetic and environmental influences on stages of alcohol use across adolescence and into young adulthood. Behav Genet 2006;36:483-97.

145. Pandina RJ, Johnson V. Familial drinking history as a predictor of alcohol and drug consumption among adolescent children. J Stud Alcohol 1989;50:245-53.

146. Peck SC, Vida M, Eccles JS. Adolescent pathways to adulthood drinking: sport activity involvement is not necessarily risky or protective. Addiction 2008;103 (Suppl 1):69-83.

147. Percy A, Iwaniec D. The validity of a latent class typology of adolescent drinking patterns. Irish Journal of Psychological Medicine. 2007;24:13-8.

148. Percy A, Thornton M, McCrystal P. The extent and nature of family alcohol and drug use: findings from the belfast youth development study. Child Abuse Review. 2008;17:371-86.

149. Pitkanen T, Kokko K, Lyyra A-L, Pulkkinen L. A developmental approach to alcohol drinking behaviour in adulthood: a follow-up study from age 8 to age 42. Addiction. 2008;103:48-68.

150. Poikolainen K, Tuulio-Henrikkson A, Aalto-Setala T, Marttunen M, Lonnqvist J. Predictors of alcohol intake and heavy drinking in early adulthood: A 5-year follow-up of 15-19-year-old Finnish adolescents. Alcohol Alcohol 2001;36:85-8.

151. Prior M, Sanson A, Smart D, Oberklaid F. Psychological disorders and their correlates in an Australian community sample of preadolescent children. J Child Psychol Psychiatry 1999;40:563-80.

152. Pulkkinen L. Youthful smoking and drinking in a longitudinal perspective. J Youth Adolesc 1983;12:253-83.

153. Pulkkinen L, Pitkanen T. A prospective study of the precursors to problem drinking in young adulthood. J Stud Alcohol 1994;55:578-87.

154. Pulkkinen L, Lyyra A-L, Kokko K. Life success of males on nonoffender, adolescence-limited, persistent, and adult-onset antisocial pathways: Follow-up from age 8 to 42. Aggress Behav 2009;35:117-35.

155. Rantakallio P. Family background to and personal characteristics underlying teenage smoking. Background to teenage smoking. Scand J Soc Med 1983;11:17-22.

156. Rew L, Horner SD, Brown A. Health-risk behaviors in early adolescence. Issues Compr Pediatr Nurs. 2011;34:79-96.

157. Rhee SH, Hewitt JK, Young SE, Corley RP, Crowley TJ, Stallings MC. Genetic and environmental influences on substance initiation, use, and problem use in adolescents. Arch Gen Psychiatry. 2003;60:1256-64.

158. Roche KM, Ahmed S, Blum RW. Enduring consequences of parenting for risk behaviors from adolescence into early adulthood. Social Sci Med 2008;66:2023-34.

159. Rodgers-Farmer AY. Parental monitoring and peer group association. Journal of Social Service Research. 2001;27:1-18.

160. Roosa MW, Tein JY, Groppenbacher F, Michaels M, Dumka R. Mothers' Parenting Behavior and Child Mental Health in Families with a Problem Drinking Parent. Journal of Marriage and Family. 1993;55:107-18.

161. Rose RJ, Kaprio J, Winter T, Koskenvuo M, Viken RJ. Familial and socioregional environmental effects on abstinence from alcohol at age sixteen. J Stud Alcohol Suppl 1999;13:63-74.

162. Rossow I, Kuntsche E. Early onset of drinking and risk of heavy drinking in young adulthood-A 13-year prospective study. Alcohol Clin Exp Res 2013;37(Suppl 1):E297-E304.

163. Schacht PM, Cummings EM, Davies PT. Fathering in family context and child adjustment: a longitudinal analysis. J Fam Psychol 2009;23:790-7.

164. Schmid B, Laucht M. Factors predisposing to early onset of tobacco use. Kindheit Und Entwicklung 2008;17:14-21.

165. Schmid B, Blomeyer D, Treutlein J, Zimmermann US, Buchmann AF, Schmidt MH, et al. Interacting effects of CRHR1 gene and stressful life events on drinking initiation and progression among 19-year-olds. Int J Neuropsychopharmacol 2010;13:703-14.

166. Scholes-Balog KE, Hemphill S, Reid S, Patton G, Toumbourou J. Predicting early initiation of alcohol use: a prospective study of Australian children. Subst Use Misuse 2013;48:343-52.

167. Scholte RH, Poelen EA, Willemsen G, Boomsma DI, Engels RC. Relative risks of adolescent and young adult alcohol use: the role of drinking fathers, mothers, siblings, and friends. Addicti Behav 2008;33:1-14.

168. Seljamo S, Aromaa M, Koivusilta L, Rautava P, Sourander A, Helenius H, et al. Alcohol use in families: A 15-year prospective follow-up study. Addiction 2006;101:984-92.

169. Sellers CS, Winfree Jr LT. Differential associations and definitions: A panel study of youthful drinking behavior. Int J Addict 1990;25:755-71.

170. Shucksmith J, Glendinning A, Hendry L. Adolescent drinking behaviour and the role of family life: A Scottish perspective. J Adolesc 1997;20:85-101.

171. Siebenbruner J, Englund MM, Egeland B, Hudson K. Developmental antecedents of late adolescence substance use patterns. Dev Psychopathol 2006;18:551-71.

172. Sieving RE, Maruyama G, Williams CL, Perry CL. Pathways to Adolescent Alcohol Use: Potential Mechanisms of Parent Influence. Journal of Research on Adolescence 2000;10:489-514.

173. Simons-Morton B. Prospective association of peer influence, school engagement, drinking expectancies, and parent expectations with drinking initiation among sixth graders. Addict Behav 2004;29:299-309.

174. Simons-Morton B. Social influences on adolescent substance use. American Journal of Health Behavior. 2007;31:672-84.

175. Skeer M, McCormick MC, Normand SL, Buka SL, Gilman SE. A prospective study of familial conflict, psychological stress, and the development of substance use disorders in adolescence. Drug Alcohol Depend 2009;104:65-72.

176. Sobeck J, Abbey A, Agius E, Clinton M, Harrison K. Predicting early adolescent substance use: Do risk factors differ depending on age of onset? J Subst Abuse 2000;11:89-102.

177. Sorensen HJ, Manzardo AM, Knop J, Penick EC, Madarasz W, Nickel EJ, et al. The contribution of parental alcohol use disorders and other psychiatric illness to the risk of alcohol use disorders in the offspring. Alcohol Clin Exp Res 2011;35:1315-20.

178. Sourander A, Elonheimo H, Niemela S, Nuutila AM, Helenius H, Sillanmaki L, et al. Childhood predictors of male criminality: A prospective population- based follow-up study from age 8 to late adolescence. J Am Acad Child Adolesc Psychiatry 2006;45:578-86.

179. Spein AR, Sexton H, Kvernmo SE. Longitudinal Drinking Patterns in Indigenous Sami and Non-Indigenous Youth in Northern Norway. J Ethn Subst Abuse 2006;5:103-17.

180. Stanton WR, Silva PA. A longitudinal study of the influence of parents and friends on children's initiation of smoking. Journal of Applied Developmental Psychology. 1992;13:423-34.

181. Stanton WR, Flay BR, Colder CR, Mehta P. Identifying and predicting adolescent smokers' developmental trajectories. Nicotine Tob Res 2004;6:843-52.

182. Steele RG, Forehand R, Armistead L, Brody G. Predicting alcohol and drug use in early adulthood: the role of internalizing and externalizing behavior problems in early adolescence. Am J orthopsychiatry. 1995;65:380-8.

183. Steinberg L, Fletcher A, Darling N. Parental monitoring and peer influences on adolescent substance use. Pediatr 1994;93:1060-4.

184. Stewart C, Power TG. Identifying patterns of adolescent drinking: A tri-ethnic study. J Stud Alcohol 2002;63:156-68.

185. Stice E, Barrera M, Jr., Chassin L. Prospective differential prediction of adolescent alcohol use and problem use: Examining the mechanisms of effect. J Abnorm Psychol;107:616-28.

186. Stoolmiller M, Wills TA, McClure AC, Tanski SE, Worth KA, Gerrard M, et al. Comparing media and family predictors of alcohol use: A cohort study of US adolescents. BMJ Open 2012;2:e000543.

187. Suzuki K, Takeda A, Matsushita S, Higuchi S, Shirakura K. [A cohort study of Japanese adolescent alcohol use and misuse (1): Observation for 2 years]. Nihon Arukoru Yakubutsu Igakkai Zasshi 2002;37:577-85.

188. Suzuki R, Ye W, Rylander-Rudqvist T, Saji S, Colditz GA, Wolk A. Alcohol and postmenopausal breast cancer risk defined by estrogen and progesterone receptor status: a prospective cohort study. J Ntl Cancer Inst 2005;97:1601-8.

189. Suzuki K, Matsushita S, Kimura M, Takeda A, Higuchi S. [Results of 10-year cohort study on Japanese adolescent drinking]. Nihon Arukoru Yakubutsu Igakkai Zasshi 2011;46:470-85.

190. Thomas G, Reifman A, Barnes GM, Farrell MP. Delayed onset of drunkenness as a protective factor for adolescent alcohol misuse and sexual risk taking: A longitudinal study. Deviant Behavior. 2000;21:181-210.

191. Tildesley EA, Andrews JA. The development of children's intentions to use alcohol: direct and indirect effects of parent alcohol use and parenting behaviors. Psychol Addict Behav 2008;22:326-39.

192. Ting TT, Chen WJ, Chen YY, Chen KH, Lin KM, Chen CY. Contextual, genetic, and cognitive effects on alcohol consumption during the transition into adolescence. Comprehensive Psychiatry Conference: 102nd Annual American Psychopathological Association Meeting on "Multigenerational Transmission of Psychopathology" New York, NY United States Conference Start. 2013;54(1).

193. Trim RS, Schuckit MA, Smith TL. Predicting drinking onset with discrete-time survival analysis in offspring from the San Diego prospective study. Drug Alcohol Depend 2010;107:215-20.

194. Turner AP, Larimer ME, Sarason IG. Family risk factors for alcohol-related consequences and poor adjustment in fraternity and sorority members: Exploring the role of parent-child conflict. J Stud Alcohol 2000;61:818-26.

195. Urberg K, Goldstein MS, Toro PA. Supportive relationships as a moderator of the effects of parent and peer drinking on adolescent drinking. Journal of Research on Adolescence. 2005;15:1-19.

196. van der Vorst H, Engels RCME, Meeus W, Dekovic M. The impact of alcohol-specific rules, parental norms about early drinking and parental alcohol use on adolescents' drinking behavior. J Child Psychol Psychiatry 2006;47:1299-306.

197. Van Der Vorst H, Engels RCME, Meeus W, Dekovic M, Van Leeuwe J. Similarities and bi-directional influences regarding alcohol consumption in adolescent sibling pairs. Addict Behav 2007;32:1814-25.

198. Van Der Vorst H, Burk WJ, Engels RC. The role of parental alcohol-specific communication in early adolescents' alcohol use. Drug Alcohol Depend 2010;111:183-90.

199. Van Der Zwaluw CS, Scholte RHJ, Vermulst AA, Buitelaar JK, Verkes RJ, Engels RC. Parental problem drinking, parenting, and adolescent alcohol use. J Behav Med 2008;31:189-200.

200. Van Ryzin MJ, Fosco GM, Dishion TJ. Family and peer predictors of substance use from early adolescence to early adulthood: An 11-year prospective analysis. Addict Behav 2012;37:1314-24.

201. Van Zundert RM, Van Der Vorst H, Vermulst AA, Engels RC. Pathways to alcohol use among Dutch students in regular education and education for adolescents with behavioral problems: the role of parental alcohol use, general parenting practices, and alcohol-specific parenting practices. J Fam Psychol 2006;20:456-67.

202. Vergés A, Sher KJ. Personality and contextual factors in college students' drinking. . In: White HR, Rabiner DL, editors. College drinking and drug use 2012. p. 140-58.

203. Vicary JR, Lerner JV. Parental attributes and adolescent drug use. J Adolesc1986;9:115-22.

204. Visser L, de Winter AF, Vollebergh WA, Verhulst FC, Reijneveld SA. The impact of parenting styles on adolescent alcohol use: the TRAILS study. Eur Addict Res 2013;19:165-72.

205. von Borczyskowski A, Lindblad F, Vinnerljung B, Reintjes R, Hjern A. Familial factors and suicide: an adoption study in a Swedish National Cohort. Psychol Med 2011;41:749-58.

206. Walls TA, Fairlie AM, Wood MD. Parents do matter: a longitudinal two-part mixed model of early college alcohol participation and intensity. J Stud Alcohol Drugs 2009;70:908-18.

207. Warner LA, White HR. Longitudinal effects of age at onset and first drinking situations on problem drinking. Subst Use Misuse 2003;38:1983-2016.

208. Webb JA, Baer PE. Influence of family disharmony and parental alcohol use on adolescent social skills, self-efficacy, and alcohol use. Addict Behav 1995;20:127-35.

209. Webb JA, Bray JH, Getz JG, Adams G. Gender, perceived parental monitoring, and behavioral adjustment: influences on adolescent alcohol use. Am J Orthopsychiatry 2002;72:392-400.

210. Weill J, Le Bourhis B. Factors predictive of alcohol consumption in a representative sample of French male teenagers: a five-year prospective study. Drug Alcohol Depend 1994;35:45-50.

211. Wells JE, Horwood LJ, Fergusson DM. Drinking patterns in mid-adolescence and psychosocial outcomes in late adolescence and early adulthood. Addiction 2004;99:1529-41.

212. Westling E, Andrews JA, Hampson SE, Peterson M. Pubertal timing and substance use: the effects of gender, parental monitoring and deviant peers. J Adolesc Health 2008;42:555-63.

213. Widom CS, White HR, Czaja SJ, Marmorstein NR. Long-term effects of child abuse and neglect on alcohol use and excessive drinking in middle adulthood. J Stud Alcohol Drugs 2007;68:317-26.

214. Williams B, Studies AIoF, Melbourne Uo, Trust RER, Project AT. Patterns and Predictors of Teenagers' Use of Licit and Illicit Substances in the Australian Temperament Project Cohort: University of Melbourne; 2000.

215. Wills TA, Vaccaro D, McNamara G, Hirky AE. Escalated substance use: a longitudinal grouping analysis from early to middle adolescence. J Abnorm Psychol 1996;105:166-80.

216. Windle M. An alcohol involvement typology for adolescents: convergent validity and longitudinal stability. J Stud Alcohol 1996;57:627-37.

217. Windle M, Davies PT. Depression and heavy alcohol use among adolescents: concurrent and prospective relations. Dev Psychopathol 1999;11:823-44.

218. Winqvist S, Jokelainen J, Luukinen H, Hillbom M. Parental alcohol misuse is a powerful predictor for the risk of traumatic brain injury in childhood. Brain Inj 2007;21:1079-85.

219. Wong MM, Nigg JT, Zucker RA, Puttler LI, Fitzgerald HE, Jester JM, et al. Behavioral control and resiliency in the onset of alcohol and illicit drug use: a prospective study from preschool to adolescence. Child Dev 2006;77:1016-33.

220. Wood MD, Read JP, Mitchell RE, Brand NH. Do parents still matter? Parent and peer influences on alcohol involvement among recent high school graduates. Psychol Addict Behav 2004;18:19-30.

221. Wu P, Bird HR, Liu X, Fan B, Fuller C, Shen S, et al. Childhood depressive symptoms and early onset of alcohol use. Pediatr 2006;118:1907-15.

222. Yang S, Kramer MS. Paternal alcohol consumption, family transition and child development in a former Soviet country. Int J Epidemiol 2012;41:1086-96.

223. Yen JY, Yen CF, Chen CC, Chen SH, Ko CH. Family factors of internet addiction and substance use experience in Taiwanese adolescents. Cyberpsychol Behav 2007;10:323-9.

224. Yu J. The association between parental alcohol-related behaviors and children's drinking. Drug Alcohol Depend 2003;69:253-62.

225. Zhang L, Wieczorek WF, Welte JW. The influence of parental and peer drinking behaviors on underage drinking and driving by young men. J Drug Educ 2012;42:347-59.

226. Zhang L, Welte JW, Wieczorek WF. The influence of parental drinking and closeness on adolescent drinking. J Stud Alcohol 1999;60:245-51.

227. Zhang L, Welte JW, Wieczorek WF. Peer and parental influences on male adolescent drinking. Subst Use Misuse 1997;32:2121-36.
