# Supplementary material for: Parental drinking and adverse outcomes in children: A scoping review of cohort studies
Source: Drug Alcohol Rev. 2015 Aug 31;35(4):397–405. doi: 10.1111/dar.12319 (PMC4950034; doi:10.1111/dar.12319)
Supplement: Supplementary file 2 — Table S2. Overview of studies, grouped by type of outcome. [file DAR-35-397-s002.docx]

**Appendix 2**

Table S2: Overview of studies, grouped by type of outcome

| Study number, (first) author, year | Sample type and size | Follow-up years, country | Exposure: type, time frame, categories, by whom, child’s age, reported by | Outcome(s): measure(s), child’s age(s) | Main findings, estimates, type of analysis | Study focus |
| --- | --- | --- | --- | --- | --- | --- |
| **Studies with drinking behaviour as outcome** | | | | | | |
| Adalbjarnar-dottir, 2001 [1*]* | School students, n=1293 | 3 years , Iceland | Drinking frq, no TF, 2 cat, parents comb, age 14, CR | Alcohol use (usually 5+), at age 17 | No association, adjusted for co-variates | AMF |
| Adalbjarnar-dottir, 2002 [2*]* | School students, n=1293 | 3 years, Iceland | Drinking frq, no TF, 2 cat, parents comb, at age 14, CR | Alcohol use (usually 5+), at age 17 | Parental drinking predicted alcohol use only among those w/anti-social behaviour, (RR= 3.1), adjusted for co-variate | AMF |
| Alati, 2008 [3*]* | Birth cohort, n=4363 | 9 years, Australia | Usual quantity 3+ (2 cat), only mother before pregnancy (no TF)/during pregnancy and at age 5 (no TF), PR | Alcohol use frequency/ quantity, at age 14 | Mothers’ drinking usual 3+ before, during, and after pregnancy predicted child’s greater alcohol use (various estimates for various combinations), adjusted for co-variates. | PF |
| Alati, 2014 [4] | Birth cohort, n= 751 | 4 years, Australia | Drinking categories, no TF, 5 cat, separate, at age 13.5, PR | Drinking trajectories, at ages 13.5, 15.5, and 17.5 | Increased maternal (OR=2.77, *P*<0.001) and paternal (OR=1.40, *P*=0.020) drinking at 13.5 years predicted a higher trajectory group, adjusted for co-variates | PF |
| Andrews, 1993 [5] | Community sample, n=657 | 1 year, USA | Drinking frq, no TF, 7 cat, separate, at age 11-15, PR | Alcohol use frequency, at age 12-16 | Maternal drinking predicted alcohol use only in younger girls (B=0.49, *P*<0.05), and older boys (B=1.09, *P* <0.001), adjusting for co-variates | PF |
| Armstrong, 2013 [6] | Community sample, n=374 | Min 6 years, USA | Usual quantity, no TF, 8 cat, parents comb, across ages 4.5 and 8, PR | Alcohol use trajectories, ages 14 through 17 | Parental alcohol use predicted increased initial drinking (B=0.25, *P*<0.01), effect persisted over time, adjusted for co-variates | AMF |
| Ary, 1993 [7] | Community sample, n=173 | 1 year, USA | Drinking frq, no TF, 7 cat, combined, age 11-17, PR | Alcohol use frequency, at age 12- 18 years | Parent alcohol use predicted change in alcohol use, adjusted association in path model (path coeff=0.160, *P*<0.05) | AMF |
| Bailey, 2006 [8] | Community sample, n=208 | Min 1 year, USA | Binge drinking (5+) frq (G1), no TF, 3 cat, parents comb, age 13-14, PR | Binge drinking (5+) frq, ages 15-18, 21- 24, and 27 | Parental 5+ drinking predicted offspring 5+ drinking at21-24 (χ^2^=5.64; *P* <0.05), but not at 15-18 and 27, in bi-variate analysis | PF |
| Brook, 1986 [9] | High school students, n=318 | 2 years, USA | Alcohol use, not specified, no TF, separate, age 14-15, CR | Alcohol initiation at age 16-18 | Paternal, but not maternal drinking was positively correlated with alcohol initiation r= 0.10, *P* <0.05), in bi-variate analysis | AMF |
| Burk, 2011 [10] | Community sample, n=362 | 7 years, USA | Quantity per day, no TF, 17 cat, parents comb, ages 4 and 8-9 years, PR | Usual number of drinks/occasion, in grade 10 | No association between parental drinking and adolescent drinking, adjusted for co-variates | AMF |
| Casswell, 2002 [11] | Birth cohort, n=714 | Min 9 years, New Zealand | Drinking frq and quantity, no TF, 2 cat (above vs below median), separate, at age 9, PR | Typical amount and frq of drinking trajectories, age 18 through 26 | Father’s drinking frq associated w/ higher drinking trajectories for men (OR=1.6, *P*=0.057) and mother’s drinking for women (OR=1.8, *P*=0.023), adjusted for co-variates | AMF |
| Collins, 2007 [12] | School students, n=1664 | 1 year, USA | Drinking not specified, no TF, parents comb, at age 11.8, CR | Beer drinking and drinking intentions at age 12.8 | Parental alcohol use did not predict beer drinking or drinking intentions, adjusted for co-variates | Co-variate |
| Cortes, 2009 [13] | School students, n=792 | 1-4 years, USA | Alcohol use, not specified, TF past 30 days, mother only, age 8-12, PR | Alcohol use frequency growth, age 13-17 | Maternal alcohol use predicted growth in child alcohol use (β=0.10, *P*<0.05), adjusted for co-variates | AMF |
| Donovan, 2011 [14] | Community sample, n=393 | 5.5 years (appr.), USA | Drinking frq, TF past 6 months, parents comb, at age 10, PR | Age at drinking initiation, early onset before 15 | Average parental drinking frq predicted early onset of drinking (OR=1.007, *P*<0.05), adjusted for co-variates | AMF |
| Duncan, 2011 [15] | Community sample, n=256 | 6 years, USA | Drinking frq, no TF, 9 cat, parents comb, at ages 13, 15, PR | Drinking frequency at ages 18, 20 | More frequent parental drinking predicted increased youth drinking over time (B=0.10, *P*<0.05), adjusted for co-variates. | AMF |
| Eitle, 2010 [16] | School students, n=523 | Min 7 years, USA | Immediate family troubles from alcohol, no TF, 2 cat, at age 19-21, CR | Heavy episodic drinking (HED) at age 26-31 | Family troubles due to alcohol did not predict HED, adjusted for co-variates | AMF |
| Ellickson, 1991 [17] | School students, n=1966 | 1 year, USA | Alcohol use, not specified, no TF, adult most important, at age 13, CR | Drinking frq and binge drinking (3+) frq, at age 14 (Grade 8) | Adult alcohol use did not predict drinking frequency, but binge drinking among non-users at baseline (β=0.11, *P*<0.05), adjusted for co-variates. | AMF |
| Engels, 1999 [18] | School students, n=1063 | 2 - 3 years, Netherlands | Drinking frq, no TF, 7 cat, parents comb, ages 12, 14, CR | Drinking frequency at ages 14, 17 | Parental drinking frq predicted adolescent drinking frq (B=0.130, *P*<0.001) in structural model. | PF |
| Engels, 2003 [19] | School students, 958 | 3 years, Netherlands | Drinking frq, no TF, 7 cat, combined, at age 14, CR | Drinking behaviour, drunkenness, 6+ frq, age 17 | Parental drinking predicted drinking frq (B=0.27, *P*<0.001); quantity (B=0.17, *P*<0.001); drunkenness frq (B=0.23, *P*<0.001); and 6+ frq (B=0.13, *P*<0.01), adjusted for adolescent drinking age 14 | PF |
| Fergusson, 1995 [20] | Birth cohort, n=953 | 3-5 years, New Zealand | Typical weekly volume, no TF, parents comb, at age 11, PR | Largest amount/ occasion at 14, latent measure hazardous drinking at age 16 | Parental drinking did not predict drinking at age 14 in bi-variate analysis, and was not directly associated with hazardous drinking at age 16, in recursive path model | AMF |
| Fisher, 2007 [21] | Community sample, n=5511 | 1 year, USA | Any alcohol use, no TF, 2 cat, adults in household, age 11-18, CR | Alcohol initiation and drinking 5+, at age 12-19 | Parental alcohol use predicted alcohol initiation (OR=1.82 for girls, 1.43 for boys, *P*<0.05), but not drinking 5+, adj for co-variates | AMF |
| Getz, 2005 [22] | Students, n= 3675 | 2 years, USA | Drinking frq, no TF, 5 cat, mothers only, age 11-14, CR | Experimental, moderate, and heavy alcohol use at age 13-16 | Mother’s alcohol use predicted experimental/moderate use (structure coefficient=0.32, *P*<0.001) and heavy alcohol use (structure coefficient = 0.12, *P*<0.05) two years later, adjusted for co-variates | AMF |
| Gleiberman, 1991 [23] | Community sample, n=190 | 17 years, USA | Drinking level, mixed TF, 5 cat, separate, mean age 28, PR | Drinking level at mean age 45 | Parental abstention predicted abstention in adult offspring 17 years later (Tau-B=0.21, *P*<0.001), bi-variate analyses | PF |
| Green, 1991 [24] | Birth cohort, n= 726 | 1 year, UK | Drinking categories, TF = past week, 4 cat, parents comb, age 15-16, PR | Drinking vs abstention, at age 16-17 | No overall association, but positive association in females (OR=2.57, *P*<0.01) and non-manual households (OR=3.20, *P*<0.01), adjusted for co-variate | PF |
| Hops, 1996 [25] | Community sample, N=517 | 1 year, USA | Drinking frq, mixed TF, 7 cat, separate, at age 15-19, PR | Adolescent alcohol use, at age 16-20 | Only mother’s drinking predicted adolescent alcohol use (β=.037, *P*<0.05), adjusted for co-variates | PF |
| Hung, 2009 [26] | Student sample, n= 1183 | 1 year, Taiwan | Drinking frq, mixed TF, 4 cat, parents , comb, at age 10-11, PR | Initiation alcohol use at age 11-12 | Parental alcohol use predicted child’s initiation of alcohol use one year later (OR=1.64, *P*<0.05), adjusted for co-variates | AMF |
| Jackson, 1997 [27] | Student sample, n=488 | 2 years, USA | Alcohol use, no TF, 2 cat, parents comb, at age 10-11, CR | Alcohol use, at age 12-13 | Parental alcohol use did not predict child’ alcohol use, adjusted for co-variates | AMF |
| Latend-resse, 2008 [28] | Cohort twins, n=4731 | 3-6 years, Finland | Drinking frq, intoxication frq, TF = current, 9 cat, parents comb, at age 11-12, PR | Adolescent drinking behaviour at ages 14 and 17.5 | Parental drinking behaviours predicted child’s alcohol use and intoxication at ages 14 and 17 (8 path coefficients, range 0.02 to 0.16, *P*<0.001 for all), adjusted for co-variates | PF |
| Lo, 2010 [29] | Community sample, n=3807 | 2 years, USA | Amount/day, TF = last 30 days, 3 cat, only mother, age 10-14,PR | Onset of drinking, age 10-14 | Maternal daily drinking predicted early onset of drinking (OR=1.077, *P*<0.05), adjusted for co-variates | PF |
| Marsden, 2005 [30] | School students, n= 467 | 1.5 years, UK | Drinking frq, no TF, 7 cat, separate, at age 15-16, CR | Drinking frequency and drinking intensity, at age 17 | No association with drinking frq, mother’s drinking predicted drinking intensity (B=0.153, *P*<0.01), reversed association for paternal drinking (B=-0.121, *P*<0.05), adjusted for co-variates | AMF |
| Otten, 2008 [31] | Community sample, n=404 (families) | 2 years, Netherlands | Amount per week, TF=last 7 days, sum-score, separate, age 13-15/14-17, PR | Weekly alcohol consumption, age 15-17/ 16-19 | Paternal drinking predicted only youngest child’s drinking (β=0.11, *P*<0.05), maternal drinking predicted younger (β=0.10, *P*<0.05) and older (β=0.12, *P*<0.01) siblings’ drinking, in cross-lagged path model | PF |
| Pedersen, 1998 [32] | School students, n= 465 | 6 years, Norway | Alcohol use not specified, no TF, 9 cat, parents comb, age 12-15, CR | Alcohol debut at 13-16 onwards, alcohol consumption at age 18-21 | Parental alcohol use predicted early alcohol debut (direct path effects reported as identical for boys and girls: = -0.029, *P*<0.001), not consumption at age 18-21, in path model | AMF |
| Pedersen, 2013 [33] | School students, n=2558 | Min 5 years, Norway | Drinking frq, 13 cat, binge drinking, 13 cat, no TF, parents comb, age 13-21, CR | Alcohol consumption and binge drinking at mean age 28 | Parental drinking predicted alcohol consumption (B=.09, *P*<0.01) and binge drinking (B=.10, *P*<0.01), adj for co-variates | PF |
| Peterson, 1994 [34] | School sample, n=450 | 2 years, USA | Drinking frq, no TF, 4 cat, parents comb, age 12-13, PR | Alcohol use at age 14-15 | Parental drinking did not predict alcohol use, adjusted for co-variates | PF |
| Poelen, 2007 [35] | Twin families, n= 1779 | 2-7 years, Netherlands | Drinking frq, no TF, 3 cat, separate, at age 12-25, PR | Regular drinking at ages 14-27, 19-32 | Maternal drinking few times/week predicted regular drinking 2 (OR=1.37, *P*<0.05) and 7 years (OR=1,78, *P*<0.001) later, paternal drinking daily predicted regular drinking only 2 years later (OR=1.48, *P*<0.05), adj for co-variates | AMF |
| Power, 2005 [36] | School students, n=743 | 2 years, USA | Drinking and drunkenness frq, TF = past year and past month, separate, age 15-16 (?), CR | Changes in drinking clusters, from 15-16 to 17-18 (?) | Parental drinking did not predict changes in drinking clusters, adjusted for co-variates | AMF |
| Reifman, 1998 [37] | Community sample, n= 546 | 1 year, USA | Drinking frq and amount, no TF, 6 cat, mother only, at ages 13-16 and 14-17, PR | Regular drinking, HED, at ages 14-17 and 15-18 | Maternal drinking did not predict regular drinking, but predicted HED 1 year later (OR=1.21, *P*<0.06) but not 2 years later, not clear if adjusted for co-variates | AMF |
| Saraceno, 2012 [38] | Birth cohort, n=4220 | 4 years, UK | Daily alcohol intake, no TF, separate, at age 8 or 10, PR | Alcohol use at age 14 | Maternal, not paternal alcohol use predicted adolescent alcohol use (OR=1.24, *P*=0.002 in males), (OR=1.20, *P*<0.001 in females), adj for co-variates | Co-variate |
| Spijkerman, 2007 [39] | High school students, n=1956 | 1 year, Netherlands | Drinking frq, no TF, 8 cat, parents comb, 12-14, CR | Alcohol use at age 13-15 | No direct association between parental alcohol use and child alcohol use, in SEM | AMF |
| Tyler, 2006 [40] | Youth cohort, n=244 | 4-6 years, USA | Binge drinking (5+), TF=past 30 days, 2 cat, mother only, at age 10-12, PR | Binge drinking (5+) at ages 14-16, 16-18 | Mother’s binge drinking predicted binge drinking at age 14-16 (β=.171, *P*<0.01), but not at age 16-18, adjusted for co-variates | AMF |
| Van der Vorst, 2006 [41] | Community sample, n=832 | 1 year, Netherlands | Drinking frq, volume, no TF, 6 cat, separate, ages 13.4, 15.2, PR | Drinking frequency, drinks/ week, ages 14.4 , 16.2 | Only mother’s alcohol use predicted only older adolescents’ drinking 1 year later (standardised path estimate=.12), in SEM | PF |
| Van der Vorst, 2009 [42] | Community sample, n=808 | 1-2 years, Netherlands | Volume, TF=past week, separate, ages 13, 16, PR | Consumption trajectories, 13 through and 16 through 15 and 18 | Only paternal drinking predicted drinking trajectory (various estimates for various trajectories and younger vs older adolescent), adj for co-variates | AMF |
| Van der Vorst, 2010 [43] | Community sample, n=802 | 1-2 years, Netherlands | Drinking frq, volume, TF=past week, parents comb, ages 13.4, 15.2, PR | Alcohol use at home/outside home, ages 14-17, 15-18. | Parental drinking predicted drinking at home for older (β=.168, *P*<0.05) and younger sibling (β=.155, *P*<0.05) and outside home for older (β=.118, *P*<0.05) and younger sibling (β=.156, *P*<0.05), in path model | AMF |
| Van der Vorst, 2013 [44] | High school students, n=608 | 1 year, Canada | Alcohol use categories, no TF, 7 cat, parents comb, age 11-17, CR | Alcohol use, at age 12-18 | Parental alcohol use predicted onset of adolescent alcohol use (standardised probit regression coefficient=.11, *P*<0.05), in path model | PF |
| Vermeulen-Smit, 2012 [45] | Data from RCT, n=2038 | 3 years, Netherlands | Drinking frq, volume, separate, age 12-15, PR | Drinking initiation, drinking frq, volume, trajectories from 12-15 to 15-18 | Heavy drinking father or two heavy episodic drinking parents predicted early and heavier adolescent drinking (10 of 18 standardised regr coeff stat sign), adjusted for co-variates | PF |
| Webb, 1991 [46] | School students, n=104 | 1.3 years, USA | Drinking frq, no TF, 10 cat, parents comb, at age 13, CR | Initiation of alcohol use at age 14 | Parental drinking did not predict initiation of alcohol use, in bi-variate analysis | AMF |
| Webster, 1989 [47] | Community sample, n= 420 | 17 years, USA | Volume, no TF, separate, at mean age 16-17, PR | Alcohol amount per week at mean age 33-34 (SD 8.4-10.0) | Father’s drinking (partial r=0.15, *P*=0.05) and mother’s drinking (partial r=.16, *P*=0.04) predicted alcohol use in sons, only father’s drinking (partial r=.29, *P*<0.001) predicted alcohol use in daughters, adj for co-variate. | PF |
| **Studies with alcohol-related harm as outcome** | | | | | | |
| Alati, 2005 [48] | Birth cohort, n=2386 | 7 years, Australia | Drinking frq, no TF, 4 cat, only mother, at age 14, PR | Alcohol abuse/ dependence, at age 21 | Maternal daily drinking predicted alcohol problems risk for males only, adjusted for co-variates. OR=2.04, *P*=0.017 | AMF |
| Guo, 2001 [49]) | School students, n=808 | Min 5 years, USA | Alcohol use, not specified, no TF, parents comb, at ages 10, 14, and 16, PR | Alcohol abuse/ dependence at age 21 | Only parental alcohol use at age 16 predicted alcohol abuse (OR=1.42, *P*<0.01) and alcohol dependence (OR=1.65, *P*<0.01), adjusted for co-variates | AMF |
| Maldonado-Molina, 2011 [50] | Community sample, n= 9559 | 6-7 years, USA | Drinking frq, no TF, 2 cat, parents comb, age 11-19, PR | Driving under the influence (DUI), age 18-26 | Parental alcohol use predicted DUI in women (OR=1.39, *P*<0.05) and men (OR=1.33, *P*<0.05), adjusted for co-variates | PF |
| Poelen, 2009 [51] | Twin families, n=1796 | 7 years, Netherlands | Drinking frq, no TF, 3 cat, separate, at age 12-25, PR | Problem drinking (CAGE), at age 19-32 | Only paternal drinking a few times/week predicted problem drinking 7 years later (OR=1.78, *P*<0.05), mixed findings for parental drinking and problem drinking 2 years later: father’s drinking (OR=2.24, *P*<0.01), mother’s drinking (OR=0.67, *P*<0.05), adjusted for co-variates | AMF |
| **Studies with drinking behaviour and alcohol-related harm as outcomes** | | | | | | |
| Ellickson, 2001 [52] | School students, n= 1198 - 2726 | 5 years, USA | Drinking frq, no TF, 4 cat, adult most important, at age 13, CR | Problem-related drinking, high risk drinking, high consumption, at age 18 | Adult alcohol use predicted problem-related drinking (OR=1.14, *P*<0.05), but not the two other outcomes at age 18, adjusted for co-variates | AMF |
| Englund, 2008 [53) | Birth cohort, n=162 | 7-12 years, USA | Drinking frq, no TF, 6 cat, only mother, at age 16, CR | Heavy alcohol use, ages 19/23/ 26, alcohol use disorders, age 28 | Maternal alcohol use predicted heavy use only at age 26 and only for males (OR=1.75, *P*<0.05), in bi-variate analyses | AMF |
| Fergusson, 1994 [54] | Birth cohort, n=990 | 4 years, New Zealand | Typical weekly volume, no TF, parents comb, age 11, PR | Drinking frq, typical amount, alcohol related problems, age 15 | Parental drinking did not predict any outcome measures, adjusted for co-variates | Co-variate |
| Hawkins, 1997 [] | Students, n=757 | 7 years, USA | Drinking frq, no TF, parents comb, age 10-11, PR | Alcohol initiation, alcohol misuse, at age 17-18 | Parental drinking predicted earlier drinking initiation (β=-0.19, *P*<0.05), no direct association with alcohol misuse at 17-18, in path model | AMF |
| Little, 2012 [56] | Birth cohort, n=941 | 2 years, Australia | Alcohol use categories, no TF, 5 cat, fathers only, at age 17-18, PR | Risky drinking alcohol related harms at age 19-20 | Paternal drinking predicted alcohol related harms (B=0.23, *P*<0.05), but not risky drinking 2 years later, adjusted for co-variates | AMF |
| Little, 2013 [57] | Birth cohort, n= 941 | 2 years, Australia | Alcohol use categories, no TF, 5 cat, separate, age 17-18, PR | Alcohol-related harms and risky drinking, at age 19-20 | Paternal and not maternal drinking predicted alcohol related harms (standardised path loading =0.08, *P*<0.05) and risky drinking (standardised path loading = 0.07, *P*<0.05) in path model | AMF |
| Mares, 2011 [58] | Families, n= 428 | 4 years, Netherlands | Drinking frq: 6 cat, volume, TF= past 4 weeks, separate, at age 13-16, PR | Excessive alcohol use, alcohol related problems, at age 17-20 | Paternal, but not maternal, drinking predicted only excessive drinking (β=0.16 for older and β=0.17 for younger adolescents, *P*<0.05), adjusted for co-variates | PF |
| Ouellette, 1999 [59] | Community sample, n=357 | 2 years, USA | Problem drinking, TF=current and past 3 months, mean score, parents comb, age 14-16, PR | Alcohol consumption, alcohol related problems, age 16-18 | Parental problem drinking did not predict alcohol consumption or related problems directly alcohol expectancy, in path model | AMF |
| Pedersen, 1996 [60] | School sample, n=249 | Min 1 year, Norway | Drinking frq, no TF, 10 cat, parents comb, at mean age 14, CR | Alcohol use at mean ages 15 - 20, alcohol related probl, age 20 | No direct effect of parental drinking on alcohol use or related problems, in path models | AMF |
| Thompson, 1987 [61] | School students, n=839 | 4 years, USA | Drinking frq, no TF, 3 cat, separate, at age 12-14, CR | Drinking behaviour and drinking problems at age 16-18 | Paternal drinking predicted one of four drinking outcomes in both boys (Beta=.11, *P*<0.001) and girls (Beta=.06, *P*<0.05), maternal drinking predicted one drinking outcome only in girls (Beta=.06, *P*<0.01), adjusted for co-variates | PF |
| Van den Eijnden, 2011 [62] | School students, n=537 | 2 years, Netherlands | Drinking frq and volume, no TF, parents comb, at age 12-17, CR | Alcohol consumption and problems, age 14-19 | Parental drinking did not predict alcohol consumption or alcohol related problems 2 years later, adjusted for co-variates | PF |
| VanVoorst, 2003 [63] | College students, n=169 | 3 months, USA | Alcohol problem severity, TF= life time, 20 cat, parents comb, at age 18, CR | Increase in alcohol consumption/related problems, 3 months later | Parental alcohol problems interacted w/students’ alcohol expectancies to predict increased alcohol problems (interaction term *B*=.03, *P*<0.001), adjusted for co-variates | PF |
| **Studies with other substance use/related harm as outcome** | | | | | | |
| Alati, 2008 [64] | Birth cohort, n=2286 | 16 years, Australia | Drinking frq, no TF, 3 cat, only mother, age 5, PR | Ecstasy use disorder at age 21 | No association, in bi-variate analysis | Co-variate |
| Fergusson, 2008 [65] | Birth cohort, n=900 | Min 5 years, New Zealand | Alcohol problems, life time TF, 2 cat, parents comb, at age 11, PR | Illicit drug use/dependence at ages 16-25 | Parental alcohol problems did not predict illicit drug use or dependence, adjusted for co-variates | AMF |
| Guxens, 2007 [66] | School students, n=1056 | 3 years, Spain | Any alcohol use, not specified, no TF, parents comb, at ages 12., 13.5, 14.5, CR | Onset of cannabis use between ages 13.5 and 15.5 | Parental alcohol use did not predict subsequent onset of cannabis use, adjusted for co-variates | AMF |
| Hayatbakhsh, 2007 [67] | Birth cohort, n= 3176 | 7-16 years, Australia | Frequent/high consumption, no TF, 2 cat, only mother, ages 5, 14, PR | Occasional and frequent cannabis use, age 21 | Maternal drinking at age 5 and/or at age 14 did not predict cannabis use at age 21, adjusted for co-variates | PF |
| Hayatbakhsh, 2009a) [68] | Birth cohort, n= 2332 | 7 years, Australia | Volume (1+ drink/day), no TF, 2 cat, mothers only, at age 14, PR | Cannabis use and cannabis use disorder, at age 21 | Maternal alcohol use did not predict cannabis use/disorder, adjusted for co-variates | AMF |
| Hayatbakhsh, 2009b) [69] | Birth cohort, n= 2042 | 7 years, Australia | Drinking categories, no TF, 3 cat, mothers only, at age 14, PR | Amphetamine use and disorders at age 21 | Maternal alcohol use did not predict amphetamine use/-disorder, adjusted for co-variates | AMF |
| Hayatbakhsh, 2013a) [70] | Birth cohort, n= 3007 | 16 years, Australia | Drinking categories, no TF, 3 cat, mothers only, at age 5, PR | Age of onset cannabis use, at age 21 | Maternal drinking predicted early onset cannabis use (OR=1.69, *P*<0.05), adjusted for co-variates | AMF |
| Hayatbakhsh, 2013b) [71] | Birth cohort, n=3039 | 16 years, Australia | Drinking categories, no TF, 3 cat, mothers only, at age 5, PR | Age of onset smoking, at age 21 | Maternal drinking predicted early onset smoking (HR=1.21, *P*<0.05), adjusted for co-variates | AMF |
| Korhonen, 2008 [72] | Cohort twins, n= 3118 | 6 years, Finland | Intoxication frequency (5+), no TF, 3 cat, separate, at age 11-12, PR | Lifetime cannabis/ other illicit drug use at age 17.5 | Only father’s weekly drinking to intoxication predicted illicit drug use 6 years later (OR=3.41, *P*<0.05), adjusted for co-variates | AMF |
| Menezes, 2007 [73] | Birth cohort, n=2718 | Min 14 years, Brazil | Alcohol related problems, no TF, 2 cat, fathers only, age 2-4,PR | Smoking at age 18 | Paternal alcohol related problems predicted daily (OR=4.43, *P*<0.05) and weekly (OR=3.15, *P*<0.05) smoking, adjusted for co-variates | AMF |
| O’Callag-han, 2006 [74] | Cohort mothers, n= 4541 | 9 years, Australia | Any drinking, no TF, 2 cat, mothers only, at age 5, PR | Smoking Age 14 | Maternal drinking predicted smoking (OR=1.5, *P*<0.05), adjusted for co-variates | Co-variate |
| **Studies with outcome other than substance use** | | | | | | |
| Balsa, 2009 [75] | Community sample, n=7212 | Min 9 years, USA | Alcoholic or problem drinking, no TF, 2 cat, separate, at age 23-31, CR | Self-perceived health and mental health problems at age 40 | Paternal problem drinking predicted poor mental health in women (prop score=1.47, *P*<0.05), maternal problem drinking predicted poor self-perceived health (prop score=0.21, *P*<0.05) and poor mental health (prop score=2.79, *P*<0.05) in women, adjusted for co-variates | PF |
| Brook, 2010 [76] | School sample, n= 660 | 10 years, USA | Alcohol use, not specified, no TF, separate and parents comb, age 12-16, CR | Psychological symptoms, at ages 17-21 and 22-26 | No direct effect of parental drinking on psychological symptoms at T2 or T3, in path model | PF |
| Chatterji, 2001 [77] | Community samples, n=7546 | Min 2 years, USA | Drinking frq, past month, binge drinking, past month, 2 cat, only mother, at age 4-14, PR | Problem Behaviour Index, at age 4-14 | Maternal drinking frequency (child specific fixed effect=0.104), but not binge drinking, predicted child’s problem behaviour, adjusted for co-variates | PF |
| Homish, 2010 [78] | Community sample, n=259 | 2 years, USA | Intoxication frq, 6+ frq, TF past year, 9 cat, separate, age 4-11, both CR and PR | Internalizing symptoms/ behaviour, at age 6-13 | Mother’s heavy drinking predicted internalizing symptoms in GEE model (Risk ratio=1.16; *P*<0.01) , adjusted for co-variates | AMF |
| Kahn, 2005 [79] | Community sample, n=2520 | Min 2 years, USA | Binge drinking, not specified, 4 cat, mothers only, at ages 0/6, 2/8, PR | Child behaviour problems at age 4-10 | Maternal drinking did not predict child behaviour problems, adjusted for co-variates | AMF |
| Kim, 2006 [80] | High school students, n= 11321 | 7 years, USA | Alcohol use not specified, no TF, parents comb, at age 12-18, PR | Contact with parents at age 19-25 | Parental alcohol use predicted no contact with parents 7 years later (OR=1.12, *P*<0.05), adjustment for co-variates not clear | AMF |
| Klinteberg, 2011 [81] | Birth cohort, n=14294 | Min 1 year, Sweden | Alcohol abuse, not specified, no TF, 2 cat, fathers only, at age 0-13, PR | Criminality, at age 13-27, mortality age at age 28-56 | Paternal alcohol abuse predicted criminality in males (OR=1.77, *P*<0.05) and females (OR=2.06, *P*<0.05) and mortality only in males (OR=1.92, *P*<0.05), adjusted for co-variates | AMF |
| Van der Molen, 2011 [82] | Community sample females, n=1942 | 1 year, USA | Problem drinking, mothers only, ages 7/8 - 10/11, PR | Disruptive behaviour, ages 8/9 - 11/12 | Maternal problem drinking did not predict disruptive behaviour, adjusted for co-variates | AMF |
| Van der Molen, 2012 [83] | Community sample females, n=1909 | 2 years, USA | Problem drinking, AUDIT score, TF= current, mothers only, 7-8/9-10, PR | Disruptive behaviour, ages 9-10/11-12 | High alcohol use did not predict disruptive behaviour 2 years later, adjusted for co-variates | AMF |
| **Studies with several types of outcome** | | | | | | |
| Barnes, 1994 [84] | Community sample, n=318 | 1yr, USA | Heavy drinking, TF past year, 2 cat, parents comb, age 13-16, PR | Frequent drinking 5+, drunkenness, deviance, at age 14-17 | Parental heavy drinking did not predict frequent heavy drinking or drunkenness, but number of deviant acts (F=3.3, *P*<0.05), adj for co-variates | AMF |
| Barnes, 2006 [85] | Community sample, n=506 | Min 1 year, USA | Alcohol abuse, TF past year, 2 cat, parents comb, at age 13-16, PR | Alcohol misuse, illicit drug use, delinquency, at ages 14.5 through 19.9 | No main effects of exposure on any outcome, but interacted w/ family support and peer deviance in predicting delinquency (interaction term b=-0.60, *P*=0.006), adjusted for co-variates | Co-variate |
| Cohen, 1994 [86] | School students, n=1376 | 1- 4 years, USA | Alcohol use, not specified, no TF, 2 cat (above or below average), parents comb, ages 10-14, CR | Onset smoking, onset alcohol use, ages 11-15 | Parental alcohol use predicted onset of alcohol use (RR=1.27 from 7^th^ to 8^th^ grade and RR=1.38 from 8^th^ to 9^th^ grade, *P*<0.05), not smoking, adjusted for co-variates | AMF |
| Dishion, 1999 [87] | Community sample, males, n=206 | Min 1 year, USA | Drinking 5+ frq, no TF, parents comb, at age 10-11, PR | Onsets of alcohol use, smoking, and marijuana, ages 11 - 16 | Parental drinking did not predict onset of any outcome measures, adjusted for co-variates | AMF |
| Ennett, 2001 [88] | Community sample, n=537 | 1 year, USA | Drinking, not specified, no TF, 2 cat, parents comb, at age 12-14, PR | Alcohol and cigarette initiation and escalation, at age 13-15 | Parental drinking predicted only smoking escalation (OR=2.85, *P*<0.01), adjusted for co-variates | Co-variate |
| Farrell, 1995 [89] | Community sample, n=658 | 1 year, USA | Problem drinking, TF past year, 2 cat, only father, at age 13-16, PR | Psychological distress, deviant behaviour, frequent drinking at age 14-17 | Paternal problem drinking predicted distress (B=-0.06, *P*<0.10), deviance (B=-0.28, *P*< and drinking when family cohesion was low, adjusted for co-variates | PF |
| Haugland, 2012 [90] | Students, n= 2399 | 4 years, Norway | Intoxication frq, mixed TF, 4 cat, parents comb, at age 14, CR | Frequent drinking, intoxication, illicit drug use, at age 18 | Frequent parental intoxication predicted frequent drinking (OR=3.8, *P*<0.001); repeated intoxication (OR=6.5, *P*<0.001); and illicit drug use (OR=3.0, *P*<0.001), adjusted for co-variates | PF |
| Hayatbakhsh, 2008 [91] | Birth cohort, n=3647 | 7 years, Australia | Any alcohol intake, no TF, 2 cat, only mother, at age 5, PR | Early onset alcohol, cigarettes, cannabis. Nicotine-, alcohol-, and cannabis use disorder, age 21 | Maternal alcohol use at age 5 predicted onset of smoking (OR=1.3, *P*<0.05) and cannabis use (OR=1.5, *P*<0.05), early onset of alcohol use (OR=8.5, *P*<0.05), and alcohol use disorder at age 21 (OR=1.4, *P*<0.05), adjusted for co-variates | AMF |
| Kerr, 2012 [92] | Community sample, n=125 | Min 1 year, USA | Alcohol use, average of 3 items scales, no TF, separate, at ages 5-13, PR | Externalising behaviour, alcohol use, at ages 5-13 | Mother’s (β=0.20, *P*<0.05) and father’s (β=0.22, *P*<0.10) alcohol use predicted alcohol use, not externalising behaviour, in path model | AMF |
| Li, 20026 [93] | School students, n=1551 | 1.5 years, USA | Parental alcohol use, TF=current, 3 cat, parents comb, at age 12-13, CR | Adolescent alcohol use, smoking, and marijuana use 1.5 years later | Both parents’ drinking predicted adolescent alcohol use (OR=1.9, *P*<0.05), but not smoking or marijuana use, adjusted for co-variates | PF |
| Macleod, 2008 [94] | Birth cohort, n=4064 | Min 6 years, UK | Parental drinking, no TF, 3 cat, separate, age 0-4 years, PR | Alcohol use, smoking, at age 10 | Maternal drinking predicted only alcohol use (OR=2.6, *P*<0.01) , no association with paternal drinking, adjusted for co-variates | AMF |
| Mason, 2007 [95] | Community sample, n=286 | 7 years, USA | Problem drinking, TF= past 12 months, comb, age 11, PR | Alcohol use at age 13 and 16, problem substance use at age 18 | Parental problem drinking predicted alcohol use at age 13 (estimate not reported) but not alcohol use at age 16 and problem substance use, adjusted for co-variates | Co-variate |
| Pears, 2007 [96] | Families, n=103 | 7 years, USA | Drinking frq, no TF, parents comb, at age 9-10, PR/CR | Alcohol use frequency, discipline at age 16-18 | Grandparents’ alcohol use predicted parents’ alcohol use (path coefficient = 0.22, *P*<0.05) and poor discipline (path coefficient=0.19, *P*<0.05), in path model | PF |
| Sieber 1990 [97] | Cohort male conscripts, n=3155 | 12 years, Switzerland | Drinking frq, intoxication frq, no TF, 5 cat, separate, at age 19, CR | Alcohol use, tobacco use, cannabis use, at age 31 | Both parents drinking/intoxication predicted alcohol -, tobacco -, and cannabis use (12 of 24 corr coeff stat sign), in bi-variate analyses | AMF |
| White, 2000 [98] | Community sample, n=432 | 13 years, USA | Drinking frq, no TF, 9 cat, separate, at age 15, CR | Heavy drinking, heavy smoking trajectories from age 15 to age 28 | Mother’s drinking predicted heavy drinking trajectories in females (OR=3.0, *P*<0.05) and males (OR=2.9, *P*<0.05), father’s drinking predicted heavy drinking trajectory in males (OR=2.8, *P*<0.05), not clear whether adjusted for co-variates | PF |
| Windle, 2000 [99] | Students, n= 975 (?) | 0.5-1 year, USA | Volume, TF=last 30 days, separate, at age 16 (?), PR | Alcohol use, alcohol problems, illicit drug use, at age 17 | Parental alcohol use did not predict adolescents’ alcohol use, adjusted for co-variates | AMF |

*Note*: sample size is the number of persons in the (multi-variate) analysis.

AMF, among multiple factors; cat, number of categories; CR, child report; frq, frequency; HED, heavy episodic drinking; parents comb, measure of parental drinking combined; OR, odds ratio; PF, primary focus; PR, parental report; SEM, structural equation modeling; TF, time frame.

**References**

**References**

1. Adalbjarnardottir S, Rafnsson FD. Perceived control in adolescent substance use: concurrent and longitudinal analyses. Psychol Addict Behav 2001;15:25–32.

2. Adalbjarnardottir S, Rafnsson FD. Adolescent antisocial behavior and substance use: longitudinal analyses. Addict Behav 2002;27:227–40.

3. Alati R, Clavarino A, Najman JM, O’Callaghan M, Bor W, Mamun AA, *et al*. The developmental origin of adolescent alcohol use: findings from the Mater University Study of Pregnancy and its outcomes. Drug Alcohol Depend 2008;98:136–43.

4. Alati R, Baker P, Betts K, Connor J, Little K, Sanson A, *et al*. The role of parental alcohol use, parental discipline and antisocial behaviour on adolescent drinking trajectories. Drug Alcohol Depend 2014;134:178–84.

5. Andrews JA, Hops H, Ary D, Tildesley E, Harris J. Parental influence on early adolescent substance use specific and nonspecific effects. J Early Adolesc 1993;13:285–310.

6. Armstrong JM, Ruttle PL, Burk LR, Costanzo PR, Strauman TJ, Essex MJ. Early risk factors for alcohol use across high school and its covariation with deviant friends. J Stud Alcohol Drugs 2013;74:746.

7. Ary DV, Tildesley E, Hops H, Andrews JA. The influence of parent, sibling, and peer modeling and attitudes on adolescent use of alcohol. Subst Use Misuse 1993;28:853–80.

8. Bailey JA, Hill KG, Oesterle S, Hawkins JD. Linking substance use and problem behavior across three generations. J Abnorm Child Psychol 2006;34:263–82.

9. Brook JS, Whiteman M, Gordon AS, Nomura C, Brook DW. Onset of adolescent drinking: a longitudinal study of intrapersonal and interpersonal antecedents. Adv Alcohol Subst Abuse. 1986;5:91–110.

10. Burk LR, Armstrong JM, Goldsmith HH, Klein MH, Strauman TJ, Costanzo PR, *et al*. Sex, temperament, and family context: how the interaction of early factors differentially predict adolescent alcohol use and are mediated by proximal adolescent factors. Psychol Addict Behav 2011;25:1.

11. Casswell S, Pledger M, Pratap S. Trajectories of drinking from 18 to 26 years: identification and prediction. Addiction 2002;97:1427–37.

12. Collins RL, Ellickson PL, McCaffrey D, Hambarsoomians K. Early adolescent exposure to alcohol advertising and its relationship to underage drinking. J Adolesc Health 2007;40:527–34.

13. Cortes RC, Fleming CB, Mason WA, Catalano RF. Risk factors linking maternal depressed mood to growth in adolescent substance use. J Emotion Behav Disord 2009;17:49– 64.

14. Donovan JE, Molina BSG. Childhood risk factors for early-onset drinking. J Stud Alcohol Drugs 2011;72: 741.

15. Duncan SC, Gau JM, Duncan TE, Strycker LA. Development and correlates of alcohol use from ages 13–20. J Drug Educ 2011;41:235–52.

16. Eitle D, Taylor J, Eitle TM. Heavy episodic alcohol use in emerging adulthood: the role of early risk factors and young adult social roles. J Drug Issues 2010;40:295–320.

17. Ellickson PL, Hays RD. Antecedents of drinking among young adolescents with different alcohol use histories. J Stud Alcohol Drugs 1991;52:398.

18. Engels RCME, Knibbe RA, Vries HD, Drop MJ, Breukelen GJP. Influences of parental and best friends’ smoking and drinking on adolescent use: a longitudinal study1. J Appl Soc Psychol 1999;29:337–61.

19. Engels RCME, van der Vorst H. The roles of parents in adolescent and peer alcohol consumption. Neth J Soc Sci 2003.

20. Fergusson DM, Horwood LJ, Lynskey MT. The prevalence and risk factors associated with abusive or hazardous alcohol consumption in 16-year-olds. Addiction 1995;90:935–46.

21. Fisher LB, Miles IW, Austin SB, Camargo CA, Colditz GA. Predictors of initiation of alcohol use among US adolescents: findings from a prospective cohort study. Arch Pediatr Adolesc Med 2007;161:959–66.

22. Getz JG, Bray JH. Predicting heavy alcohol use among adolescents. Am J Orthopsychiatry 2005;75:102–16.

23. Gleiberman L, Harburg E, Di Franceisco W, Schork A. Familial transmission of alcohol use: IV. A seventeen-year follow-up on the relationships between parent and adult offspring alcohol use;Tecumseh, Michigan. Int J Epidemiol 1991;20:441–7.

24. Green G, Macintyre S, West P, Ecob R. Like parent like child? Associations between drinking and smoking behaviour of parents and their children. Br J Addict 1991;86:745–58.

25. Hops H, Duncan TE, Duncan SC, Stoolmiller M. Parent substance use as a predictor of adolescent use: a six-year lagged analysis. Ann Behav Med 1996;18:157–64.

26. Hung C-C, Yen L-L, Wu W-C. Association of parents’ alcohol use and family interaction with the initiation of alcohol use by sixth graders: a preliminary study in Taiwan. BMC Public Health 2009;9:172.

27. Jackson C. Initial and experimental stages of tobacco and alcohol use during late childhood: relation to peer, parent, and personal risk factors. Addict Behav 1997;22:685–98.

28. Latendresse SJ, Rose RJ, Viken RJ, Pulkkinen L, Kaprio J, Dick DM. Parenting mechanisms in links between parents’ and adolescents’ alcohol use behaviors. Alcohol Clin Exp Res 2008;32:322–30.

29. Lo CC, Cheng TC. Onset drinking: how it is related both to mother’s drinking and mother-child relationships. Subst Use Misuse 2010;45:888–900.

30. Marsden J, Boys A, Farrell MP, Stillwell G, Hutchings K, Hillebrand J, *et al*. Personal and social correlates of alcohol consumption among mid-adolescents. Br J Dev Psychol 2005;23:427–50.

31. Otten R, Van Der Zwaluw CS, Van Der Vorst H, Engels RCME. Partner effects and bidirectional parent-child effects in family alcohol use. Eur Addict Res 2008;14:106–12.

32. Pedersen W, Skrondal A. Alcohol consumption debut: predictors and consequences. J Stud Alcohol 1998;59:32–42.

33. Pedersen W, von Soest T. Socialization to binge drinking: a population-based, longitudinal study with emphasis on parental influences. Drug Alcohol Depend 2013;133:587– 92.

34. Peterson PL, Hawkins JD, Abbott RD, Catalano RF. Disentangling the effects of parental drinking, family management, and parental alcohol norms on current drinking by black and white adolescents. J Res Adolesc 1994;4:203–27.

35. Poelen EAP, Scholte RHJ, Willemsen G, Boomsma DI, Engels RCME. Drinking by parents, siblings, and friends as predictors of regular alcohol use in adolescents and young adults: a longitudinal twin-family study. Alcohol Alcohol 2007;42:362–9.

36. Power TG, Stewart CD, Hughes SO, Arbona C. Predicting patterns of adolescent alcohol use: a longitudinal study. J Stud Alcohol Drugs 2005;66:74–81.

37. Reifman A, Barnes GM, Dintcheff BA, Farrell MP, Uhteg L. Parental and peer influences on the onset of heavier drinking among adolescents. J Stud Alcohol Drugs 1998;59:311.

38. Saraceno L, Heron J, Munafò M, Craddock N, van den Bree M. The relationship between childhood depressive symptoms and problem alcohol use in early adolescence: findings from a large longitudinal population-based study. Addiction 2012;107:567–77.

39. Spijkerman R, Van den Eijnden RJJM, Overbeek G, Engels RCME. The impact of peer and parental norms and behavior on adolescent drinking: The role of drinker prototypes. Psychol Health 2007;22:7–29.

40. Tyler KA, Stone RT, Bersani B. Examining the changing influence of predictors on adolescent alcohol misuse. J Child Adolesc Subst Abuse 2006;16:95–114.

41. Van DerVorst H, Engels RCME, Meeus W, Dekovic´ M. The impact of alcohol-specific rules, parental norms about early drinking and parental alcohol use on adolescents’ drinking behavior. J Child Psychol Psychiatry 2006;47:1299–306.

42. Van Der Vorst H, Vermulst AA, Meeus WH, Dekovic´ M, Engels RC. Identification and prediction of drinking trajectories in early and mid-adolescence. J Clin Child Adolesc Psychol 2009;38:329–41.

43. Vorst HVD, Engels RCME, Burk WJ. Do parents and best friends influence the normative increase in adolescents’ alcohol use at home and outside the home? J Stud Alcohol Drugs 2010;71:105.

44. Van Der Vorst H, Krank M, Engels RCME, Pieters S, Burk WJ, Mares SHW. The mediating role of alcohol-related memory associations on the relation between perceived parental drinking and the onset of adolescents’ alcohol use. Addiction 2013;108:526–33.

45. Vermeulen-Smit E, Koning IM, Verdurmen JEE, Van der Vorst H, Engels RCME, Vollebergh WAM. The influence of paternal and maternal drinking patterns within two-partner families on the initiation and development of adolescent drinking. Addict Behav 2012;37:1248–56.

46. Webb JA, Baer PE, Caid CD, McLaughlin RJ, McKelvey RS. Concurrent and longitudinal assessment of risk for alcohol use among seventh graders. J Early Adolesc 1991;11:450–65.

47. Webster DW, Harburg E, Gleiberman L, Schork A, DiFranceisco W. Familial transmission of alcohol use: I. Parent and adult offspring alcohol use over 17 years— Tecumseh, Michigan. J Stud Alcohol Drugs 1989;50:557.

48. Alati R, Najman JM, Kinner SA, Mamun AA, Williams GM, O’Callaghan M, e*t al.* Early predictors of adult drinking: a birth cohort study. American J Epidemiol 2005;162:1098– 107.

49. Guo J, Hawkins JD, Hill KG, Abbott RD. Childhood and adolescent predictors of alcohol abuse and dependence in young adulthood. J Stud Alcohol 2001;62:754.

50. Maldonado-Molina MM, Reingle JM, Delcher C, Branchini J. The role of parental alcohol consumption on driving under the influence of alcohol: Results from a longitudinal, nationally representative sample. Accid Anal Prev 2011;43:2182–7.

51. Poelen EAP, Engels RCME, Scholte RHJ, Boomsma DI, Willemsen G. Predictors of problem drinking in adolescence and young adulthood. Eur Child Adolesc Psychiatry 2009;18:345–52.

52. Ellickson PL, Tucker JS, Klein DJ, McGuigan KA. Prospective risk factors for alcohol misuse in late adolescence. J Stud Alcohol Drugs 2001;62:773.

53. Englund MM,Egeland B, Oliva EM, CollinsWA. Childhood and adolescent predictors of heavy drinking and alcohol use disorders in early adulthood: a longitudinal developmental analysis. Addiction 2008;103(s1):23–35.

54. Fergusson DM, Lynskey MT, Horwood LJ. Childhood exposure to alcohol and adolescent drinking patterns. Addiction 1994;89:1007–16.

55. Hawkins JD, Graham JW, Maguin E, Abbott RD, Hill KG, Catalano RF. Exploring the effects of age of alcohol use initiation and psychosocial risk factors on subsequent alcohol misuse. J Stud Alcohol 1997;58:280.

56. Little K, Hawkins MT, Sanson A, Toumbourou JW, Smart D, Vassallo S, *et al*. The longitudinal prediction of alcohol consumption-related harms among young adults. Subst Use Misuse 2012;47:1303–17.

57. Little K, Hawkins MT, Sanson A, O’Connor M, Toumbourou JW, Smart D, *et al*. Longitudinal predictors of alcohol-related harms during the transition to adulthood. Aust Psychol 2013;48:270–80.

58. Mares SHW, van der Vorst H, Engels RCME, Lichtwarck-Aschoff A. Parental alcohol use, alcohol-related problems, and alcohol-specific attitudes, alcohol-specific communication, and adolescent excessive alcohol use and alcohol-related problems: an indirect path model. Addict Behav 2011;36:209–16.

59. Ouellette JA, Gerrard M, Gibbons FX, Reis-Bergan M. Parents, peers, and prototypes: antecedents of adolescent alcohol expectancies, alcohol consumption, and alcoholrelated life problems in rural youth. Psychol Addict Behav 1999;13:183–97.

60. Pedersen W, Skrondal A. Alcohol and sexual victimization: a longitudinal study of Norwegian girls. Addiction 1996;91:565–81.

61. Thompson KM, Wilsnack RW. Parental influence on adolescent drinking: modeling, attitudes, or conflict? Youth Soc 1987;19:22–43.

62. Eijnden RVD, Van De Mheen D, Vet R, Vermulst AA. Alcohol-specific parenting and adolescents’ alcohol-related problems: the interacting role of alcohol availability at home and parental rules. J Stud Alcohol Drugs 2011;72:408.

63. VanVoorst WA, Quirk SW. Are relations between parental history of alcohol problems and changes in drinking moderated by positive expectancies? Alcohol Clin Exp Res 2003;27:25–30.

64. Alati R, Kinner SA, Hayatbakhsh MR, Mamun AA, Najman JM, Williams GM. Pathways to ecstasy use in young adults: anxiety, depression or behavioural deviance? Drug Alcohol Depend 2008;92:108–15.

65. Fergusson DM, Boden JM, Horwood LJ. The developmental antecedents of illicit drug use: evidence from a 25-year longitudinal study. Drug Alcohol Depend 2008;96:165–77.

66. Guxens M, Nebot M, Ariza C. Age and sex differences in factors associated with the onset of cannabis use: a cohort study. Drug Alcohol Depend 2007;88:234–43.

67. Hayatbakhsh MR, Alati R, Hutchinson DM, Jamrozik K, Najman JM, Mamun AA, *et al*. Association of maternal smoking and alcohol consumption with young adults’ cannabis use: a prospective study. Am J Epidemiol 2007; 166:592–8.

68. Hayatbakhsh MR, Najman JM, Bor W, O’Callaghan MJ, Williams GM. Multiple risk factor model predicting cannabis use and use disorders: a longitudinal study. Am J Drug Alcohol Abuse 2009;35:399–407.

69. Hayatbakhsh MR, Najman JM, Bor W, Williams GM. Predictors of young adults’ amphetamine use and disorders: a prospective study. Drug Alcohol Rev 2009;28:275–83.

70. Hayatbakhsh R, Williams GM, Bor W, Najman JM. Early childhood predictors of age of initiation to use of cannabis: a birth prospective study. Drug Alcohol Rev 2013;32:232–40.

71. Hayatbakhsh R, Mamun AA, Williams GM, O’Callaghan MJ, Najman JM. Early childhood predictors of early onset of smoking: a birth prospective study. Addict Behav 2013; 38:2513–9.

72. Korhonen T, Huizink AC, Dick DM, Pulkkinen L, Rose RJ, Kaprio J. Role of individual, peer and family factors in the use of cannabis and other illicit drugs: a longitudinal analysis among Finnish adolescent twins. Drug Alcohol Depend 2008;97:33–43.

73. Menezes A, Hallal PC, Horta BL. Early determinants of smoking in adolescence: a prospective birth cohort study. Cad Saúde Pública 2007;23:347–54.

74. O’Callaghan FV, O’Callaghan M, Najman JM, Williams GM, Bor W, Alati R. Prediction of adolescent smoking from family and social risk factors at 5 years, and maternal smoking in pregnancy and at 5 and 14 years. Addiction 2006;101:282–90.

75. Balsa AI, Homer JF, French MT. The health effects of parental problem drinking on adult children. J Ment Health Policy Econ 2009;12:55–66.

76. Brook JS, Balka EB, Crossman AM, Dermatis H, Galanter M, Brook DW. The relationship between parental alcohol use, early and late adolescent alcohol use, and young adult psychological symptoms: a longitudinal study. Am J Addict 2010;19:534–42.

77. Chatterji P, Markowitz S. The impact of maternal alcohol and illicit drug use on children’s behavior problems: evidence from the children of the national longitudinal survey of youth. J Health Econ 2001;20:703–31.

78. Homish GG, Edwards EP, Eiden RD, Leonard KE. Analyzing family data: A GEE approach for substance use researchers. Addict Behav 2010;35:558–63.

79. Kahn RS, Wilson K, Wise PH. Intergenerational health disparities: socioeconomic status, women’s health conditions, and child behavior problems. Public Health Rep 2005; 120:399–408.

80. Kim KJ. Parent–adolescent conflict, negative emotion, and estrangement from the family of origin. Res Human Dev 2006;3:45–58.

81. af Klinteberg B, Almquist Y, Beijer U, Rydelius P-A. Family psychosocial characteristics influencing criminal behaviour and mortality-possible mediating factors: a longitudinal study of male and female subjects in the Stockholm Birth Cohort. BMC Public Health 2011;11:756.

82. van der Molen E, Hipwell AE, Vermeiren R, Loeber R. Maternal characteristics predicting young girls’ disruptive behavior. J Clin Child Adolesc Psychol 2011;40:179–90.

83. van der Molen E, Hipwell AE, Vermeiren R, Loeber R. Cumulative effects of mothers’ risk and promotive factors on daughters’ disruptive behavior. J Abnorm Child Psychol 2012;40:727–39.

84. Barnes GM, Farrell MP, Banerjee S. Family influences on alcohol abuse and other problem behaviors among black and white adolescents in a general population sample. J Res Adolesc 1994;4:183–201.

85. Barnes GM, Hoffman JH, Welte JW, Farrell MP, Dintcheff BA. Effects of parental monitoring and peer deviance on substance use and delinquency. J Marriage Fam 2006;68:1084–104.

86. Cohen DA, Richardson J, LaBree L. Parenting behaviors and the onset of smoking and alcohol use: a longitudinal study. Pediatrics 1994;94:368–75.

87. Dishion TJ, Capaldi DM, Yoerger K. Middle childhood antecedents to progressions in male adolescent substance use an ecological analysis of risk and protection. J Adolesc Res 1999;14:175–205.

88. Ennett ST, Bauman KE, Foshee VA, Pemberton M, Hicks KA. Parent-child communication about adolescent tobacco and alcohol use: what do parents say and does it affect youth behavior? J Marriage Fam 2001;63:48–62.

89. Farrell MP, Barnes GM, Banerjee S. Family cohesion as a buffer against the effects of problem-drinking fathers on psychological distress, deviant behavior, and heavy drinking in adolescents. J Health Soc Behav 1995;36:377–85.

90. Haugland SH, Strandheim A, Bratberg G. Is high-risk use of intoxicants more common among adolescents who have seen their parents intoxicated? Nor Med J 2012;132:410–3.

91. Hayatbakhsh MR, Mamun AA, Najman JM, O’Callaghan MJ, Bor W, Alati R. Early childhood predictors of early substance use and substance use disorders: prospective study. Aust N Z J Psychiatry 2008;42:720–31.

92. Kerr DC, Capaldi DM, Pears KC, Owen LD. Intergenerational influences on early alcohol use: independence from the problem behavior pathway. Dev Psychopathol 2012;24:889–906.

93. Li C, Pentz MA, Chou C-P. Parental substance use as a modifier of adolescent substance use risk. Addiction 2002;97:1537–50.

94. Macleod J, Hickman M, Bowen E, Alati R, Tilling K, Smith GD. Parental drug use, early adversities, later childhood problems and children’s use of tobacco and alcohol at age 10: birth cohort study. Addiction 2008;103:1731–43.

95. Mason WA, Hitchings JE, Spoth RL. Emergence of delinquency and depressed mood throughout adolescence as predictors of late adolescent problem substance use. Psychol Addict Behav 2007;21:13.

96. Pears K, Capaldi DM, Owen LD. Substance use risk across three generations: the roles of parent discipline practices and inhibitory control. Psychol Addict Behav 2007;21:373.

97. Sieber MF, Angst J. Alcohol, tobacco and cannabis: 12-year longitudinal associations with antecedent social context and personality. Drug Alcohol Depend 1990;25:281–92.

98. White HR, Johnson V, Buyske S. Parental modeling and parenting behavior effects on offspring alcohol and cigarette use: a growth curve analysis. J Subst Abuse 2000;12:287–310.

99. Windle M. Parental, sibling, and peer influences on adolescent substance use and alcohol problems. Appl Dev Sci 2000;4:98–110.
